# Description of chemical systems by means of response functions

**DOI:** 10.1007/s00285-025-02191-3

**Published:** 2025-02-16

**Authors:** E. Franco, B. Kepka, J. J. L. Velázquez

**Affiliations:** 1 Institute for Applied Mathematics, University of Bonn, Endenicher Allee 60, 53115 Bonn, Germany; 2 Institute of Mathematics, University of Zürich, Winterthurerstrasse, 190 8057 Zürich, Switzerland

**Keywords:** Renewal equations, Response functions, Non-Markovian dynamics, Biochemical systems

## Abstract

In this paper we introduce a formalism that allows to describe the response of a part of a biochemical system in terms of renewal equations. In particular, we examine under which conditions the interactions between the different parts of a chemical system, described by means of linear ODEs, can be represented in terms of renewal equations. We show also how to apply the formalism developed in this paper to some particular types of linear and non-linear ODEs, modelling some biochemical systems of interest in biology (for instance, some time-dependent versions of the classical Hopfield model of kinetic proofreading). We also analyse some of the properties of the renewal equations that we are interested in, as the long-time behaviour of their solution. Furthermore, we prove that the kernels characterising the renewal equations derived by biochemical system with reactions that satisfy the detail balance condition belong to the class of completely monotone functions.

## Introduction

A basic problem in biology is to determine the response of a system (that might be a cell, a cell organelle, a specific biochemical network or a tissue) to a chemical signal. The response typically might be a chemical, electrical or mechanical output. A formalism relating the input and the output using the so-called response-time distribution has been proposed in Thurley et al. (2018). In particular, this formalism has been applied there to study signaling mechanisms between different immune cells. The goal of this paper is to formulate precise mathematical conditions which allow to use the formalism of Thurley et al. (2018) to model general biochemical systems.

In the simplest model the relation between the input signal *I*(*t*) and the output *R*(*t*) of a system is given by

1 $$\begin{aligned} \begin{aligned} \frac{dN(t) }{dt}&= R(t) \\ R\left( t\right)&=R_{0}\left( t\right) +\int _{0}^{t}\psi \left( t-s, N(s) \right) I\left( s\right) ds. \end{aligned} \end{aligned}$$

In this formula *N*(*t*) is the density of elements in the system, $R_{0}\left( t\right)$ is a transient response, associated to the initial state of the system and the integral term describes the response to the input $I\left( t\right) .$ The function $R_0$ is also called forcing function, see for instance (Diekmann et al. 1998). The input/output functions $I\left( t\right)$ and $R\left( t\right)$ might be vectors, if the system under consideration has several inputs and several outputs. Therefore, in general, $\psi$ would be a matrix.

Notice that the formalism of response functions, using equations like (1), is particularly suited to study biological systems, specifically biochemical systems. Indeed, due to the large number of substances involved in these processes, it is often difficult to determine all the reactions, as well as the relevant chemical coefficients, that would be needed to model the system in detail. On the other hand, response equations with the form (1) (or non-linear versions of it) require only the knowledge of the function $\psi$, which, in principle, can be determined experimentally from measurements of the behaviour of the system.

It is worth to mention that systems with the form (1) have been extensively used in the modeling of biological systems. The earliest example appears in population dynamics, specifically in demography, see the seminal work by Sharpe and Lotka firstly published in 1911 in Philosophical Magazine, Series, Vol.21: 435–438 and more recently published in Sharpe and Lotka (1977). Similar approaches to the one in Thurley et al. (2018) can be found in models of immune systems, see for instance (Busse et al. 2010), in models that describe the production of $Ca^{2+}$, see (Moenke et al. 2012; Thurley and Falcke 2011), in models of kinetic proofreading (Bel et al. 2009) and in models of the circadian rhythms (Thurley et al. 2017).

In this paper, motivated by the work in Thurley et al. (2018), we analyze under which conditions it is possible to study the interactions of different parts of a biochemical system by means of a set of response functions that generalizes (1). In the case of a linear system, our approach consists in considering a (large) subset of reactions as an unique object. In this paper, we call this object *compartment*. Our interest is to understand the interactions between different compartments. In fact, these interactions are described in detail by response functions $\psi$, that can be derived from the reactions taking place inside each compartment. Once the response functions have been derived, one can study the resulting response function equation and ignore the detailed information on the processes taking place inside each compartment.

We anticipate that the evolution of the number of elements $N_\alpha$ in the compartment $\alpha$ will be described, in the linear case and under suitable assumptions on the interactions between the compartments of the biochemical system, by the following system of equations

2 $$\begin{aligned} \frac{d N_\alpha (t) }{dt } = B_\alpha (t) - D_\alpha (t), \quad N_\alpha (0)=N_\alpha ^0 \end{aligned}$$

where $B_\alpha$ satisfies the renewal equation

3 $$\begin{aligned} B_\alpha (t) = B_\alpha ^0(t) + \sum _{\beta \ne \alpha } \int _0^t B_\beta (s ) \Phi _{ \beta \alpha }(t-s) ds \end{aligned}$$

and where $D_\alpha$ is a function of $B_\alpha$, namely

4 $$\begin{aligned} D_\alpha (t)=D^0_\alpha (t) + \int _0^t k_\alpha (t-s) B_\alpha (s) {ds}. \end{aligned}$$

In our study, the variable $\alpha \in X$ is usually a compartment of a larger system $\Omega$. More precisely, $\Omega$ is the set of all possible states of elements in the system. Then, *X* is a partition of $\Omega$, i.e. *X* is the set of all compartments $\alpha$. We assume that $\Omega$, and hence also *X*, is finite. In particular, (2)–(4) is a finite system of equations. In this paper we mainly study the system of Eqs. (2)–(4). However we also analyse a generalization of it, called GRFE (see Sect. 2.2) where no assumption is required on the interactions between the compartments of the network.

We will refer from now on to the Eqs. (2)–(4) as Response Function Equations (RFEs). Notice that we are assuming that the chemicals in the system are in different states and the changes in the density of elements in a certain state is only due to jumps from one state to another. Hence, in this paper we restrict ourselves to conservative system, i.e. systems for which the total number of elements is constant in time. However, it would be possible to study also non-conservative systems, similar to the ones which appear naturally in population dynamics, see e.g. Diekmann et al. (1998).

The functions $B_{\alpha },\ D_{\alpha }$ yield the total fluxes of elements from any compartment $\beta \in X$ towards $\alpha \in X, \ \alpha \ne \beta$ and from the compartment $\alpha$ towards any other compartment $\beta \in X,\ \beta \ne \alpha$ respectively. Notice that the set of functions $\left\{ B_{\alpha }\right\} ,\ \left\{ D_{\alpha }\right\}$ are related by means of input-response equations of the form (1). More precisely, a flux $B_{\beta }(s)$ arriving to the compartment $\beta \in X$ at time *s* yields a response $B_{\beta }(s)\Phi _{\beta \alpha }(t-s)$ at the compartment $\alpha$ at time *t*. The formula yielding $B_{\alpha }(t)$ in (3) takes into account the sum of the fluxes arriving to the compartment $\beta$ for all times $s\in \left( 0,t\right) .$

The function $D^0_\alpha$ is the total flux of elements, that were already in the compartment $\alpha$ at time 0, to any compartment $\beta \in X$. Similarly $B_\alpha ^0$ is the flux of elements arriving in $\alpha$ from any compartment $\beta$, given that they where in $\beta$ already at time equal zero.

Since we assume that $\sum _{\alpha \in X }N_{\alpha }$ is conserved, we assume $\sum _{\alpha \in X}D_\alpha ^0 = \sum _{\alpha \in X}B_\alpha ^0$ as well as

5 $$\begin{aligned} k_\alpha = \sum _{\beta \in X \setminus \{ \alpha \} } \Phi _{\alpha \beta }. \end{aligned}$$

Furthermore, for consistency, we need to assume

6 $$\begin{aligned} N_\alpha (0) \ge \int _0^\infty D^0_\alpha (t) dt \end{aligned}$$

for every $\alpha \in X$. This is a natural assumption that guarantees that the out-flux from the compartment $\alpha$ of the elements, which where in $\alpha$ already at time $t=0$, must be less than the total number of elements in $\alpha$ at time 0.

Finally, we assume

7 $$\begin{aligned} \sum _{\beta \in X\setminus \{\alpha \}} \int _0^\infty \Phi _{\alpha \beta }(t)\, dt=1, \end{aligned}$$

if it is possible to go from compartment $\alpha$ to any other compartment $\beta$. Otherwise, if $\alpha$ is not connected to any $\beta \in X$ then

8 $$\begin{aligned} \sum _{\beta \in X\setminus \{\alpha \}} \int _0^\infty \Phi _{ \alpha \beta }(t)\, dt = 0. \end{aligned}$$

Assumption (7) guarantees that an element moves away from a certain state in finite time with probability one.

Equations of the form (2)–(4) are one of the main objects of our study in this paper. Although in this paper we are mainly concerned with linear models, we also introduce some non-linear variants.

Let us recall that equations of the form (3) are commonly referred to as *renewal equations*, (REs). Such equations, as well as some non-linear versions of them, have been extensively studied in the mathematical literature. For instance, they have been used to analyze structured populations, see e.g. Feller (1967), Diekmann et al. (1998), Gripenberg et al. (1990), Franco et al. (2021, 2023), or epidemiological models, see e.g. Diekmann et al. (2013), Inaba (2017), Kermack and McKendrick (1927). Similar equations, in which $\Phi _{\alpha \beta }$ contain in addition a dependence on the concentrations $\left\{ N_{\gamma }\right\} _{\gamma \in \Omega }$, have been considered in Thurley et al. (2018).

It is known that linear renewal equations modelling the evolution in time of a structured population can be reformulated as a partial differential equation with a transport term and with birth-death terms. See for instance (Calsina et al. 2016). Similarly, this is possible for the RFEs (2)–(4). The corresponding PDEs contain a transport term and birth-death terms. We refer to these types of models as structured population equations (SPEs). They have the form

9 $$\begin{aligned} \begin{aligned} \partial _{t}f_{\alpha }(t,x)+\partial _{x}f_{\alpha }(t,x)&= -\Lambda _{\alpha }(x)f_{\alpha }(t,x),\ \ x\ge 0,\ \ t\ge 0,\ \ \alpha \in X \\ f_{\alpha }\left( t,0\right)&=\sum _{\beta \in X \setminus \{ \alpha \} } \int _{0}^{\infty }\lambda _{\beta \alpha }(x) f_{\beta }(t,x)dx \\ f_{\alpha }(0,x)&=f_{\alpha ,0}\left( x\right) \end{aligned} \end{aligned}$$

where $$\Lambda _{\alpha }(x):=\sum _{X\setminus \{\alpha \} } \lambda _{\alpha \beta }(x) \ge 0.$$

In the context of this paper, the family of solutions $\{ f_\alpha (t,x) \}$ of the Eq. (9) is the density of elements with state in the compartment $\alpha \in X$ and with age *x* at time *t*. The particular type of SPEs considered here is conservative, i.e. $\partial _{t}\left( \sum _{\alpha \in \Omega }\int _{0}^{\infty }f_{\alpha }(t,x)dx\right) =0$. Notice that in these equations we assume that the elements of the population with trait in $\alpha$ and age *x* are removed with rate $\Lambda _{\alpha }(x)$ and they re-appear in the population as elements with trait in $\beta \ne \alpha$ and age $x=0$ at rate $\lambda _{\alpha \beta }$.

Usually in classical structured population models the parameter *x* could be the age, the size or the immunity level against a certain pathogen, see (Diekmann et al. 1998; Franco et al. 2021). In this model *x* is the time for which an element has been in a certain compartment. Introducing the age structure allows to reduce the non-Markovian system of RFEs, with forcing function $B_\alpha ^0$ satisfying a suitable consistency condition (cf. 67), to a Markovian system of PDEs. Here with Markovian system of PDEs we mean that the transition from a certain state to another one does not depend on the history of the function $\{ f_\alpha \}$.

Let us remark that SPEs of the form (9) have been extensively used in Mathematical Biology, in particular in population dynamics, see for instance (Perthame 2006). Solutions to SPEs describe the evolution of individuals (e.g. cells, humans, animals, etc.) in a population structured via a certain variable (e.g. age, size, immunity against a certain pathogen). In this paper, we study the equivalence of a generalization of the SPE (9) with a generalization of the RFE (2)–(4), namely the GRFE (see Sect. 2.3).

Chemical systems in Systems Biology are often formulated as ODEs. Here, we denote by $\Omega$ the finite set of possible states of the elements in the system. The elements are for instance cells in different states or, alternatively, chemical substances which can be transformed in another one by means of chemical reactions.

The system is described by a finite number of concentrations $n(t)=(n_{i}(t))_{i\in \Omega }\in \mathbb {R}_{+}^{|\Omega |}$. Here, $|\Omega |\in \mathbb {N}$ denotes the total number of states. The concentrations evolve according to ODEs of the form

10 $$\begin{aligned} \dfrac{dn}{dt}(t)=An(t),\ n\left( 0\right) =n_{0}, \end{aligned}$$

where the matrix $A\in \mathbb {R}^{|\Omega |\times |\Omega |}$ satisfies

$$\begin{aligned} A_{ii}=-\sum _{k\in \Omega \backslash \{i\}}A_{ki}\ \ \text {for all }i\in \Omega \ \,\ \ A_{ki}\ge 0\ \text {for }k\ne i. \end{aligned}$$

As we will see in this paper, one can reduce a system of ODEs (10) to the RFEs (2)–(4). To this end, we partition the set of states $\Omega$ into compartments, i.e. in a family of disjoint sets $\alpha \in X$. Then, our study concerns merely the concentrations $N_{\alpha }= \sum _{k\in \alpha }n_{k}$ within each compartment $\alpha \in X$ as well as the inward and outward fluxes. Let us mention that for general choices of matrices *A* these fluxes solve more general RFEs than those in (2)–(4).

It is important to remark that, while the evolution of the solution $n\in \mathbb {R}_{+}^{|\Omega |}$ of (10) is Markovian, the evolution of $\{N_{\alpha }\}_{\alpha \in X}$ is non-Markovian. Therefore, we say that reformulating the ODEs (10) as a RFE is a *demarkovianization* process. Notice that the ODEs (10) are in general not equivalent to the RFEs (2)–(4), unless some information on the internal states of each compartment is available. However, the information on the evolution of internal states before time 0 is contained in the functions $\{D_\alpha ^0\}$ and $\{ B_\alpha ^0\}$. Similarly, the response functions $\Phi _{ \alpha \beta }(t)$ are given by the evolution of internal states at time *t*.

Once a RFE has been derived from a ODEs system, we can think of each compartment as a *black box*. The RFE model describes interactions between these black boxes. On the other hand, the concentrations $n_{k}$ can be interpreted as a set of internal variables, which characterize each compartment completely. The demarkovianization procedure yields a system with a smaller number of variables. Thus, one replaces a large Markovian system by a smaller non-Markovian system.

This procedure is reminiscent of the construction of so-called hidden Markov models (cf. Baum and Petrie (1966)). The goal in that case is to study the evolution of a Markov-process X, with unobservable (hidden) variables, by analysing only the evolution of the observable variables. We refer to Bishop and Thompson (1986) where hidden Markov processes have been applied to analyse DNA sequences for the first time.

It is relevant to mention that the demarkovianization process in this paper is different from a procedure called lumping, which has been extensively studied in chemical engineering, see (Atay and Roncoroni 2017). In lumping a large system of ODEs is reduced to a smaller system of ODEs, i.e. a Markovian process. This is only possible if the initial system of ODEs has a particular structure. In the procedure studied here the systems obtained are in general non-Markovian.

Furthermore, let us mention that ODEs of the from (10) describe pure jump Markov processes. Similarly, RFEs of the form (2)–(4) describe the so-called semi-Markov processes, see (Gyllenberg and Silvestrov 2008). These semi-Markov processes are, as well, pure jump processes. However, while in the case of Markov processes jump times are always exponentially distributed, semi-Markov processes allow for more general distributions of the jump times. In fact, the response functions $\Phi _{ \alpha \beta }$ are exactly the probability densities of these jump time distributions.

Let us now give an overview of the problems studied in this paper using the response function formalism. First of all, we study the relation between the ODEs and the RFEs in the linear case. We formulate in a precise manner the response function $\Phi _{\alpha \beta }$ corresponding to the decomposition in compartments of the systems of ODEs (10). In addition, we prove that for any set of response functions $\Phi _{\alpha \beta }$ satisfying (7) it is possible to find a sequence of ODEs with the form (10), such that the corresponding response functions converge to $\Phi _{\alpha \beta }$. In other words, we prove that the set of response functions corresponding to the class of ODEs (10) is dense in the set of probability measures (endowed with the weak topology). We refer to Sect. 3 for more details.

In this paper we consider also systems of the form (10) satisfying the detailed balance condition, i.e. we assume that at the equilibrium each individual reaction is balanced (cf. Liggett (1985)). It turns out that the class of response functions that can be derived from these systems is much smaller than the one obtained for the general systems with the form (10). More precisely, the response functions $\Phi _{\alpha \beta }$ obtained from a general class of ODEs satisfying the detailed balance condition belong to the family of completely monotone functions, i.e. they are Laplace transforms of non-negative measures (see Theorem 3.2). We remark that, to have completely monotone response functions is a necessary condition for systems originating from ODEs with detailed balance, but it is not sufficient. There are also systems of ODEs, that do not satisfy the detailed balance condition, for which the response function is completely monotone. Theorem 3.2 provides a way to discriminate these systems from the ones satisfying the detailed balance condition.

We underline that the detailed balance property is a consequence of the reversibility in time of the quantum mechanical equations (Boyd 1974). Therefore, closed biochemical systems must satisfy the detailed balance property. However, many biological systems are open and exchange substances with the environment, for instance they consume ATP molecules and release ADP molecules. The outfluxes/influxes of substances keep these systems out of equilibrium and justify the lack of detailed balance, which is typical of many biological systems. A few examples of biological systems for which it has been experimentally estabilshed that they operate out of equilibrium are molecular motors ( Alberts et al. 2002), actively beating Chlamydomonasflagella ( Battle et al. 2016) or the kinetic proofreading mechanisms (see Sect. 7.1)

As far as we know, this is the first property that has been derived for response functions that at the microscopic level are described by reactions satisfying the detailed balance property. It is relevant to mention that the characterization of biochemical systems for which the detailed balance condition holds or fails is an active research area (see for instance (Battle et al. 2016; Li et al. 2019; Martínez et al. 2019)). For instance in Martínez et al. (2019) a measure of the degree of irreversibility of general semi-Markov processes has been obtained. However, notice that the question of determining if the semi-Markov process under consideration has been obtained from a chemical system satisfying the detailed balance condition, is not addressed in Martínez et al. (2019).

Recall that the demarkovianization procedure yields a system of RFEs, which are in general non-Markovian. It is then relevant to classify the response functions for which the corresponding RFEs are actually Markovian. In fact, we prove, for a specific class of forcing function, that such response functions are exactly given by exponentials. A similar result is known for REs arising in population dynamics and in epidemiology, see (Diekmann et al. 2020; Diekmann and Inaba 2023). Furthermore, in terms of semi-Markov processes, this result is related to the fact that the only jump time distribution which yields a Markov process is the exponential distribution.

Next, we study the long-time behaviour of solutions to (2)–(4). Specifically, we give conditions on the response functions that guarantee that the solution $\{N_\alpha \}$ to (2)–(4) converge to a unique stable distribution. For this we rely on Laplace transform methods similar to the ones in Diekmann et al. (2012).

Another issue that we discuss in this paper is the reformulation of RFEs of the form (2)–(4) in terms of SPEs of the form (9). This reformulation relies on the introduction of a canonical age structure. We stress that the age structure will not be an intrinsic property of the elements of the system, but a set of auxiliary variables that allows to *Markovianize* the evolution of the densities of elements in the compartments.

In order to illustrate the use of the formalism of RFEs developed in this paper, we provide several examples of linear models, which are well-established in Biochemistry and in Systems Biology. In particular, we study the evolution in time of two variations of the classical Hopfield model (Hopfield 1974) of kinetic proofreading using the machinery of RFEs. Furthermore, we exhibit a model of polymerization combined with proofreading (see (Pigolotti and Sartori 2016)) that results in a non-Markovian polymerization rate. Then, we also formulate in terms of RFEs a linear version of the classical Barkai-Leibler model for robust adaptation (Barkai and Leibler 1997).

Finally, we consider examples of non-linear models. In this situation the class of RFEs must be much more general than the one in (1). Specifically, it would be relevant to determine under which conditions a class of RFEs is equivalent to non-linear chemical reactions with non-linearities of the kind of the mass action or more complicated ones like Michaelis-Menten or Hill’s law. In particular, the response function contains in general, multiple integrals of the form

11 $$\begin{aligned} \int _{-\infty }^t \dots \int _{-\infty }^t I(s_1) \dots I(s_n) \psi (t-s_{1}, \dots t-s_{n} ) ds_{1} \dots ds_n. \end{aligned}$$

Notice that operators with the form (11) can provide information on the time correlations of the signal *I*(*s*). In contrast, such correlations can not be described by the integral operators in (1). This is not surprising since linear ODE systems cannot yield information about time correlations of the incoming signal, in contrast with non-linear systems.

The non-linear examples discussed here include a model of non-Markovian polymerization as well as a system of ODEs describing the standard Coherent Type 1 Feed Forward Loop. The latter has been extensively considered in Systems Biology (cf. Alon (2019)).

Some of the results of this paper have been derived in different contexts in the literature, as for instance the characterization of response functions associated Markovian dynamics or the density of the response functions associated to ODEs systems in the space of probability measures. However, in order to unify the notation and to present the results in the context of chemical reactions considered in this paper, we decided to include the proofs of these results here.

### Notation and plan of the paper

Before explaining the plan of this paper let us collect here some notation used later on. First of all we define ${\mathbb {R}}_+:= [0, \infty )$ and ${\mathbb {R}}_*:= (0, \infty )$. Given a set $\Omega$, we denote with $2^\Omega$ to denote the set of all subsets of $\Omega$. We denote by $C_c({\mathbb {R}}_+)$ the space of continuous functions with compact support endowed with the supremum norm. Moreover we write ${\mathcal {M}} ({\mathbb {R}}_+)$ for the space of Radon measures on ${\mathbb {R}}_+$. We denote with ${\mathcal {M}}_{+} ({\mathbb {R}}_+)$ the cone of non-negative Radon measures and with ${\mathcal {M}}_{ b} ({\mathbb {R}}_+)$ the space of bounded Radon measures. Furthermore, we write ${\mathcal {M}}_{ +, b} ({\mathbb {R}}_+)$ to indicate the cone of non-negative bounded Radon measures. Let us recall that endowing ${\mathcal {M}}_{b}({\mathbb {R}}_+)$ with the total variation norm $\Vert \cdot \Vert _{TV}$ yields a Banach space which can be identified with $C_0({\mathbb {R}}_+)^*$. Here, $C_0({\mathbb {R}}_+)$ denotes the space of continuous functions vanishing at infinity endowed with the supremum norm.

Furthermore, let us recall that the weak convergence in the sense of measures is defined by duality with bounded continuous functions $C_b(\mathbb {R}_+)$, i.e. $\mu _n\rightharpoonup \mu$ if and only if

$$\begin{aligned} \int _{\mathbb {R}_+}\phi (x)\, \mu _n(dx) \rightarrow \int _{\mathbb {R}_+}\phi (x)\, \mu (dx) \end{aligned}$$

as $$ n\rightarrow \infty $$ for any $$ \phi \in C_b(\mathbb {R}_+) $$. Let us denote by $$d_w $$ a metric inducing weak convergence, e.g. the Lévy-Prokhorov metric.

The space $$C([0, T]; {\mathcal {M}}_{+, b}({\mathbb {R}}_+) ) $$ contains all continuous functions from [0, *T*] to $${\mathcal {M}}_{+,b}({\mathbb {R}}_+)$$. Here, we endow the space $${\mathcal {M}}_{+, b} ({\mathbb {R}}_+)$$ with the Wasserstein distance $$W_1 $$ defined by

$$\begin{aligned} W_1(\mu , \nu ):= \sup _{\{ \Vert \varphi \Vert _{Lip} \le 1 \}} \int _{{\mathbb {R}}_+} \varphi (x) (\mu - \nu )(dx) \end{aligned}$$

where the supremum is taken over the Lipschitz functions and where

$$\begin{aligned} \Vert \varphi \Vert _{Lip }= \Vert \varphi \Vert _\infty + \sup _{ \{ x,y \in {\mathbb {R}}_+, x \ne y \} } \frac{|\varphi (x) - \varphi (y) |}{|x-y |}. \end{aligned}$$

Furthermore, we denote with $L^1_{loc}({\mathbb {R}}, {\mathbb {R}}^{n \times m })$ the space of measurable functions from ${\mathbb {R}}$ to ${\mathbb {R}}^{n \times m }$ that are locally integrable and with $W^{1,1}_{loc}({\mathbb {R}}_+, {\mathbb {R}})$ the space of locally absolutely continuous functions from ${\mathbb {R}}_+$ to ${\mathbb {R}}$. Let us also define by $L^1({\mathbb {R}}_+, e^{- z_0 t } dt )$ the space of measurable functions *f* from ${\mathbb {R}}_+$ to ${\mathbb {R}}$ such that $t \mapsto f(t) e^{ z_0 t }$ is integrable.

In addition, we write $\hat{f} (z)$ for the Laplace transform of *f*, i.e.

$$\begin{aligned} \hat{f} (\lambda ):= \int _0^\infty e^{- \lambda t } f(t) dt. \end{aligned}$$

Finally, for $A\in \mathbb {R}^{n\times m}$ we write $\Vert A \Vert$ to indicate the matrix norm induced by the standard euclidean norm on $\mathbb {R}^{n}$ and $\mathbb {R}^{m}$. As no ambiguity should arise we abuse notation and write $\Vert \cdot \Vert$ for the standard euclidean norm for vectors.

The paper is organized as follows. In Sect. 2 we discuss the reformulation of a linear systems of ODEs (10) in a system of RFEs. In Sect. 3 we characterize the response functions corresponding to general linear systems of ODEs as well as systems with detailed balance. Section 4 is devoted to the characterization of response functions $\Phi _{\alpha \beta }$ in (2)–(4) corresponding to a Markovian evolution. In particular, we prove that such response functions are exactly exponentials. Then, we analyze the long-time behaviour of solutions to (2)–(4) in Sect. 5. Furthermore, the equivalence of RFEs and SPEs is discussed in Sect. 6. In Sects. 7 and 8 we present both linear and non-linear examples of biochemical systems whose response can be described using RFEs. Finally, we include some concluding remarks in Sect. 9.

## Reformulation of linear ODEs in terms of RFEs

In this section we study how to rewrite systems of ODEs of the form (10) using the RFEs formalism. More precisely, we will show that the concentrations of elements with different states in the compartment $\alpha$, $\{ n_\alpha \}$ with $n_\alpha \in {\mathbb {R}}_+^{|\alpha |}$ satisfy a system of ODEs (cf. (15)). Then we define the concentration $N_\alpha$ of individuals with states in compartment $\alpha \in X$ by

12 $$\begin{aligned} N_\alpha (t):=\sum _{j \in \alpha } (n_\alpha (t) )_j= {\textbf {e}}^\top _{|\alpha |} n_\alpha (t). \end{aligned}$$

Here, we use the notation

13 $$\begin{aligned} {\textbf {e}}_n =(1,\ldots ,1)^\top \in \mathbb {R}^{n}. \end{aligned}$$

for ${\textbf {e}}_\alpha$. As we will see, the evolution of $\{N_\alpha \}$ is given by a generalization of the RFEs described in the introduction, cf. (2)–(4), which are valid only when the compartments have at most one entrance point. In Sect. 2.1 we introduce the decomposition of the system (10) into compartments. In Sect. 2.2 we formulate and study the generalized RFEs. In Sect. 2.3, instead, we explain the relation between the system of ODEs (15) and the RFEs.

### Decomposition into compartments

In this section we introduce the decomposition of the system (10) into compartments. We also give a definition of a special class of decompositions into compartments that have at most one entrance point.

Recall that the set of the states, $\Omega$, is finite. The dynamics of concentrations $n=(n_i)_{i\in \Omega }\in \mathbb {R}_+^{|\Omega |}$ are given by

14 $$\begin{aligned} \dfrac{dn(t) }{dt}= An(t), \quad t >0. \end{aligned}$$

We assume the matrix $A\in \mathbb {R}^{|\Omega |\times |\Omega |}$ to be of the form

$$\begin{aligned} A_{ij} = \lambda _{ji}\ge 0 \text { for } i,\, j\in \Omega , \, i\ne j, \quad A_{ii} = - \sum _{k\in \Omega \backslash \{i\}} \lambda _{ik}. \end{aligned}$$

Hence, ${\textbf {e}}_{|\Omega |}^\top A =0$ where we are using the notation (13). We recall that $\lambda _{ji}$ is the jump rate from state *i* to *j*. The elements of the matrix *A* are defined as $A_{ij}=\lambda _{ji}$ instead of $A_{ij}=\lambda _{ij}$ in order to avoid to have the matrix $A^T$ in (14).

**Fig. 1:**
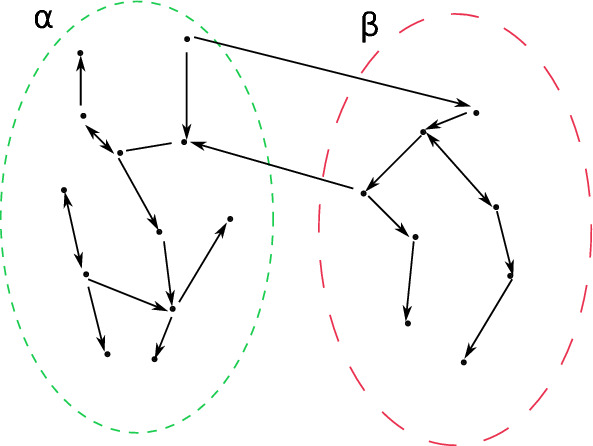
Partition of a graph in two compartments $\alpha$ and $\beta$. The straight lines connecting the elements are Markovian reactions

Let us note that the system of ODEs (14) is related to a pure Markov jump process with jump rates $\lambda _{ij}\ge 0$. Furthermore, these rates induce a graph structure on the state space $\Omega$. More precisely, we can define the set of the (directed) edges $\mathcal {E}\subset \Omega ^2$ by $\mathcal {E}=\left\{ (i,j)\in \Omega ^2 \ : \ \lambda _{ij}>0 \right\}$. Then $(\Omega , {\mathcal {E}})$ is a directed graph. See Fig. 1 for a graphical representation of the division into compartments.

For our reduction in compartments we choose a partition $X \subset 2^\Omega$ of the set $\Omega$. Accordingly, the solution of (14) can be decomposed in $n(t)=(n_\alpha (t))_{\alpha \in X }$ where $n_\alpha (t)\in \mathbb {R}_+^{|\alpha |}$ is the evolution in time of the density of elements in the compartment $\alpha \in X$. In order to specify the equations solved by $\{n_\alpha \}_{ \alpha \in X}$, we decompose the matrix *A* as

$$\begin{aligned} A = \left( \begin{matrix} A_{\alpha \beta } \end{matrix}\right) _{\alpha ,\beta \in X}. \end{aligned}$$

Here, the matrices $A_{\alpha \beta }\in \mathbb {R}^{|\alpha | \times |\beta | }$ are defined by $A_{\alpha \beta }=(A_{ij})_{i\in \alpha ,j\in \beta } = (\lambda _{ji})_{i\in \alpha ,j\in \beta }$ for every $\alpha , \beta \in X$ such that $\alpha \ne \beta$. Instead, when $\alpha =\beta$ we define

$$\begin{aligned} A_{\alpha \alpha } = E_{\alpha \alpha } - C_{\alpha }. \end{aligned}$$

where the matrices $E_{\alpha \alpha } \in {\mathbb {R}}_+^{|\alpha | \times |\alpha |}$ are given by $(E_{\alpha \alpha })_{ij} = A_{ij}=\lambda _{ji}$ when $i,j\in \alpha$, $i\ne j$. While for $i=j$ we have $(E_{\alpha \alpha })_{ii} = -\sum _{k\in \alpha }\lambda _{ik}.$ The matrix $C_{\alpha } \in {\mathbb {R}}_+^{|\alpha | \times |\alpha |}$ contains the loss terms due to the jumps from $\alpha$ to other compartments. In particular, $C_\alpha$ is a diagonal matrix of the form $(C_{\alpha })_{ii} = \sum _{j\in \Omega {\setminus } \{\alpha \}} \lambda _{ij} \text { where } i \in \alpha .$

As a consequence of (14) we have for all $n_\alpha$

15 $$\begin{aligned} \frac{d n_\alpha }{dt}(t)= E_{\alpha \alpha }n_{\alpha } (t) - C_\alpha n_\alpha (t)+ \sum _{\beta \in X \setminus \{\alpha \}} A_{\alpha \beta } n_\beta (t). \end{aligned}$$

Accordingly, the initial condition is $n_\alpha (0)=n_\alpha ^0$ for a given $n_\alpha ^0 \in {\mathbb {R}}_+^{|\alpha |}$. Finally, the graph structure $(\Omega , \mathcal {E})$ suggests the following definition.

#### Definition 2.1

(Entrance point) Consider a directed graph $(\Omega ,\mathcal {E})$ and a partition $X\subset 2^{\Omega }$. Let $\alpha \in X$. A state $i\in \alpha$ is an entrance point if there exists $\beta \in X{\setminus }\{\alpha \}$ and $j\in \beta$ such that $(j,i)\in \mathcal {E}$.

As mentioned in the introduction we sometimes restrict our attention to decompositions into compartments that have at most one entrance point. This single entrance point (if it exists) is then denoted by $i_\alpha \in \alpha$.

### Generalized RFEs

In this section we introduce the generalization of the RFE, termed GRFE. While RFEs describe the interactions between compartments that have at most one entrance point, GRFEs do not require this assumption on the compartments. The evolution in time of the concentrations of elements in the compartment $\alpha \in X$ according to the GRFE is given by

16 $$\begin{aligned} \frac{d N_\alpha }{dt }(t)&= {\textbf {e}}_\alpha ^\top S_\alpha (t) - {\textbf {e}}_\alpha ^\top J_\alpha (t), \quad N_\alpha (0) = N_\alpha ^0\ge 0, \end{aligned}$$

17 $$\begin{aligned} S_\alpha (t)&= S_\alpha ^0(t) + \sum _{\beta \in X \setminus \{\alpha \} } \int _0^t G_{\beta \alpha } (t-s) S_\beta (s) ds, \end{aligned}$$

18 $$\begin{aligned} J_\alpha (t)&= J^0_\alpha (t) + \int _0^t K_{\alpha } (t-s) S_\alpha (s) ds. \end{aligned}$$

The fluxes $\{S_\alpha (t) \}, \{\, J_\alpha (t)\}$ with $S_\alpha , J_\alpha \in \mathbb {R}_+^{|\alpha |}$ as well as the concentrations $\{ N_\alpha \}$ with $N_\alpha \in \mathbb {R}_+^{|\alpha |}$ are the unknowns. Instead, the kernels $G_{\alpha \beta }(t) \in {\mathbb {R}}_+^{|\beta | \times |\alpha |}$, $K_\alpha (t) \in {\mathbb {R}}_+^{|\alpha | \times |\alpha |}$ and the forcing functions $S_\alpha ^0(t) \in {\mathbb {R}}_+^{|\alpha | }$, $J^0_\alpha (t) \in {\mathbb {R}}_+^{|\alpha | }$ are given data.

Let us mention that (16)–(18) is a closed system of equations, so once $\{S_\alpha \}$ is known, we can deduce $\{J_\alpha \}$ and $\{ N_\alpha \}$. While the influxes and the outfluxes $\{ B_\alpha \}$ and $\{ D_\alpha \}$ in (2)–(4) are real valued functions of time, the fluxes $\{S_\alpha \}$ and $\{ J_\alpha \}$ are now vector-valued. Here, the *i*-th component of $S_\alpha$ can be interpreted as the flux of elements, coming from any other compartment, to the state $i \in \alpha$. Analogously, the *i*-th component of the vector $J_\alpha$ is the out-flux from the state $i \in \alpha$ to any state *j* in some compartment $\beta \ne \alpha$.

Concerning the well-posedness of the system (16)–(18) we rely on the following result.

#### Lemma 2.1

Assume that for all $\alpha , \beta \in X$

19 $$\begin{aligned} G_{\alpha \beta } \in L^1_{\operatorname {loc}}(\mathbb {R}_+; \mathbb {R}_+^{|\beta |\times |\alpha |}), \quad K_\alpha \in L^1_{\operatorname {loc}}(\mathbb {R}_+; \mathbb {R}_+^{|\alpha |\times |\alpha |}), \quad S^0_\alpha ,\, J^0_\alpha \in L^1_{\operatorname {loc}}(\mathbb {R}_+;\mathbb {R}_+^{|\alpha |}). \end{aligned}$$

Then, the system (16)–(18) has a unique solution with $S_\alpha , \, J_\alpha \in L^1_{\operatorname {loc}}(\mathbb {R}_+;\mathbb {R}_+^{|\alpha |})$ and $N_\alpha \in W^{1,1}_{\operatorname {loc}}(\mathbb {R}_+;\mathbb {R})$ for all $\alpha \in X$.

#### Proof

We have to prove existence and uniqueness of a solution of the renewal Eq. (17) for every $\alpha \in X$. Existence and uniqueness in $L^1_{\operatorname {loc}}$ of a solution for (17) follows from the existence of a unique resolvent $R \in L^1_{\operatorname {loc}}$, see e.g. (Gripenberg et al. (1990), Section 2.3) for the definition of resolvent and for the proof of its existence and uniqueness. The resolvent is given by an infinite series of convolutions involving the kernels, hence it is positive when the kernels are positive. This, together with the fact that the forcing functions have non-negative entries, implies that also the solution $\{S_\alpha \}, \, \{J_\alpha \}$ have non-negative entries. $\square$

The solutions to (17)–(18) do not yield, in general, non-negative concentrations $\{N_\alpha \}$. However, as we see in the following lemma, this holds under additional assumptions on the kernels and the forcing functions.

#### Lemma 2.2

Under the assumptions in Lemma 2.1 consider the unique solution of (16)–(18). Assume in addition that for all $\alpha \in X$, $j\in \alpha$

20 $$\begin{aligned} \int _0^\infty {\textbf {e}}_\alpha ^\top J_\alpha ^0(r) \, dr \le N_\alpha ^0, \quad \int _0^\infty [{\textbf {e}}_\alpha ^\top K_\alpha (r)]_j\, dr \le 1. \end{aligned}$$

Then, we have $N_\alpha (t)\ge 0$ for all $t\ge 0$, $\alpha \in X$.

#### Remark 2.1

Let us mention that condition (20) is sufficient to gurantee non-negativity of $N_\alpha$, but it is not necessary. Condition (20) appears very naturally, when we assume that the kernels $\{G_{\alpha \beta } \}, \{ K_\alpha \}$ are non zero. The first inequality in (20) ensures that the total number of elements, that where in $\alpha$ already at time zero, removed from compartment $\alpha$ does not exceed $N^0_\alpha$. The second inequality can be interpreted by viewing (16)–(18) as a semi-Markov process. The integral kernels are related to the jump probabilities. Hence, (20) ensures that the probability to leave $\alpha$ via state $j\in \alpha$ is bounded by one.

#### Proof of Lemma 2.2

According to (16), we write

$$\begin{aligned} N_\alpha (t)&= N_\alpha (0) + \int _0^t \left( {\textbf {e}}_\alpha ^\top S_\alpha (s) - {\textbf {e}}_\alpha ^\top J_\alpha (s) \right) \, ds \\&= N_\alpha (0) + \int _0^t \left( {\textbf {e}}_\alpha ^\top S_\alpha (s) - {\textbf {e}}_\alpha ^\top J_\alpha ^0(s) - \int _0^s {\textbf {e}}_\alpha ^\top K_{\alpha } (s-r) S_\alpha (r)\, dr \right) \, ds. \end{aligned}$$

Here, we used (18). We then obtain from (20)

$$\begin{aligned} N_\alpha (t)&\ge \int _0^t \left( {\textbf {e}}_\alpha ^\top S_\alpha (s) - \int _0^s {\textbf {e}}_\alpha ^\top K_{\alpha } (s-r) S_\alpha (r)\, dr \right) \, ds \\&=\int _0^t {\textbf {e}}_\alpha ^\top \left( I_{|\alpha |\times |\alpha |} - \int _s^t K_\alpha (r-s)\, dr \right) S_\alpha (s) \, ds \ge 0, \end{aligned}$$

since $(S_\alpha )_j\ge 0$ for all $\alpha \in X$, $j\in \alpha$. This concludes the proof. $\square$

We now provide some conditions ensuring the conservation of mass.

#### Lemma 2.3

Under the assumption in Lemma 2.1 consider the unique solution to (16)–(18). Assume that

21 $$\begin{aligned} \sum _{\alpha \in X} {\textbf {e}}_\alpha ^\top \left( S_\alpha ^0(t)-J^0_\alpha (t) \right) = 0, \quad \sum _{\beta \in X\setminus \{\alpha \}} {\textbf {e}}_\beta ^\top G_{\alpha \beta }(t)-{\textbf {e}}_\alpha ^\top K_\alpha (t) =0 \end{aligned}$$

for all $\alpha \in X$. Then, the total mass is conserved $\sum _{\alpha \in X}N_\alpha (t)=\sum _{\alpha \in X}N_\alpha ^0$.

#### Proof

We obtain from (16)–(18)

$$\begin{aligned} \dfrac{d}{dt}\sum _{\alpha \in X} N_\alpha (t)&= \sum _{\alpha \in X} {\textbf {e}}_\alpha ^\top \left( S_\alpha ^0(t)-J^0_\alpha (t) \right) \\&\quad + \sum _{\alpha ,\, \beta \in X, \, \alpha \ne \beta } \int _0^t {\textbf {e}}_\alpha ^\top G_{\beta \alpha }(t-s) S_\beta (s)\, ds - \sum _{\alpha \in X} \int _0^t {\textbf {e}}_\alpha ^\top K_\alpha (t-s)S_\alpha (s)\, ds \\&=\sum _{\alpha \in X}\int _0^t S_\alpha (s)\left\{ \sum _{\beta \in X\setminus \{\alpha \}} {\textbf {e}}_\beta ^\top G_{\alpha \beta }(t-s) - {\textbf {e}}_\alpha ^\top K_\alpha (t-s) \right\} \, ds =0. \end{aligned}$$

$$\square $$

### Generalized RFEs corresponding to the ODEs model

The main result of this section yields that generalized RFE, cf. (16)–(18), appear as effective equations for $\{ n_\alpha \}$ solving (15).

#### Theorem 2.4

(ODE to RFE) Consider $n(t) \in {\mathbb {R}}_+^{|\Omega |}$ solution to (14) and $N_\alpha$ defined in (12). Then, $\{N_\alpha \}$ satisfies (16). The corresponding fluxes $S_\alpha$ and $J_\alpha$ solve the system (17)–(18) with kernels

22 $$\begin{aligned} G_{\beta \alpha } (t)&= A_{\alpha \beta } e^{t A_{\beta \beta } }, \quad t \ge 0, \end{aligned}$$

23 $$\begin{aligned} \left( K_\alpha (t)\right) _{ij}&= \sum _{\beta \in X \setminus \{\alpha \}} \left( {\textbf {e}}_{\beta }^\top A_{\beta \alpha } \right) _i\left( e^{t A_{\alpha \alpha }} \right) _{ij}, \quad i,\, j\in \alpha ,\, t \ge 0, \end{aligned}$$

and forcing functions

24 $$\begin{aligned} S_\alpha ^0(t) = \sum _{\beta \in X \setminus \{\alpha \}} G_{\beta \alpha } (t) n^0_\beta ; \quad J^0_\alpha (t)= K_\alpha (t) n_\alpha ^0, \quad t \ge 0. \end{aligned}$$

Furthermore, the fluxes are given in terms of *n*(*t*) by

25 $$\begin{aligned} S_\alpha (t) = \sum _{\beta \in X \setminus \{\alpha \} }A_{\alpha \beta }n_\beta (t), \quad (J_\alpha (t))_i = (n_\alpha (t))_i\sum _{\beta \in X \setminus \{\alpha \} } \left( {\textbf {e}}_{\beta }^\top A_{\beta \alpha }\right) _i, \quad i\in \alpha \end{aligned}$$

for all $$ \alpha \in X $$.

In order to prove Theorem 2.4 we first show that the ODE (15) can be reformulated using the response function formalism by a system involving fluxes of the form $I^{i j }_{\alpha \beta }$. This term is the flux from the state $i\in \alpha$ to the state $j\in \beta$. In the proof of Theorem 2.4 below we will obtain (16)–(18) from the system solved by $\{N_\alpha \}$ and the fluxes $\{I^{i j}_{\alpha \beta }\}$.

Let us first give an informal derivation of the equations solved by $\{N_\alpha \}$ and the fluxes $\{I^{i j}_{\alpha \beta }\}$. First of all, the change of the concentration of the compartments is just due to the individual fluxes summed appropriately, thus yielding

26 $$\begin{aligned} \frac{d N_\alpha (t) }{dt } = \sum _{i \in \alpha } \sum _{\beta \in X \setminus \{\alpha \} } \sum _{j \in \beta } I_{\beta \alpha }^{ji} (t) - \sum _{i \in \alpha }\sum _{\beta \in X \setminus \{\alpha \} } \sum _{j \in \beta } I_{ \alpha \beta }^{ ij } (t). \end{aligned}$$

Here, the gain term on the right hand side involves the summation over each individual state $j\in \beta$ for some compartment $\beta \ne \alpha$ into some state $i\in \alpha$. The loss term has a similar form. Concerning the flux $I_{\alpha \beta }^{ij}$ from state $i \in \alpha$ to state $j \in \beta$ there are two contributions.

(i) One contribution is due to elements that are in $\alpha$, in some state $r\in \alpha$, already at time zero. When they evolve in time they reach state $i\in \alpha$ and jump to state $j \in \beta$ at time *t*.
(ii) The other contribution is due to the elements that jump from some state $m\in \gamma$, $\gamma \ne \alpha$ to some state $r \in \alpha$ at time $s \in (0,t)$. Then, they evolve in the compartment $\alpha$ to reach state $i \in \alpha$ and jump to state $j \in \beta$ at time *t*.

The system for the fluxes is therefore given by

27 $$\begin{aligned} I_{ \alpha \beta }^{ij} (t)=\overline{I}_{\alpha \beta }^{ij} (t) + \sum _{ r \in \alpha } \sum _{\gamma \in X \setminus \{\alpha } \}\sum _{m\in \gamma } \int _0^t \Psi _{\alpha \beta }(t-s; r, i, j ) I^{ m r }_{\gamma \alpha } (s) ds \end{aligned}$$

for all $i\in \alpha$, $j\in \beta$ and where

28 $$\begin{aligned} \overline{I}_{\alpha \beta }^{ij} (t)= \sum _{ r \in \alpha } \Psi _{\alpha \beta }(t; r, i, j) (n^0_\alpha )_r. \end{aligned}$$

The above system has again the form of RFEs. The precise form of the kernels are given in the following lemma.

Let us mention that Eqs. (26) and (27) have been formulated in the supplementary materials of Thurley and Falcke (2011).

#### Lemma 2.5

Let $n(t) \in {\mathbb {R}}_+^{|\Omega |}$ be the solution to (14) and $\{N_\alpha \}$ defined in (12). Then $\{N_\alpha \}$ satisfies (26) and the corresponding fluxes $I_{\alpha \beta }^{ij}$ are given in terms of *n*(*t*) by

29 $$\begin{aligned} I_{\alpha \beta }^{ij}(t) =(A_{\beta \alpha })_{ji} (n_\alpha (t))_i=\lambda _{ij} (n_\alpha (t))_i. \end{aligned}$$

Furthermore, the fluxes $I_{\alpha \beta }^{ij}$ solve (27) with kernels

30 $$\begin{aligned} \Psi _{\alpha \beta } (t; r , i,j)= (A_{\beta \alpha })_{ji} \left( e^{t A_{\alpha \alpha } } \right) _{ir} = \lambda _{i j } \left( e^{t A_{\alpha \alpha } } \right) _{ir} \end{aligned}$$

for $r,\, i \in \alpha$, $j \in \beta$ and forcing functions $\overline{I}_{\alpha \beta }^{ij}$ defined by (28).

#### Proof

By definition of $N_\alpha$ in (12), we have

$$\begin{aligned} \frac{d}{dt} N_\alpha (t) = {\textbf {e}}^\top _\alpha \frac{d}{dt} n_\alpha (t) = {\textbf {e}}^\top _\alpha \left( E_{\alpha \alpha } n_\alpha (t) - C_\alpha n_\alpha (t) + \sum _{\beta \in X \setminus \{\alpha \} } A_{ \alpha \beta } n_\beta (t) \right) , \end{aligned}$$

where we used (15). By definition of $E_{\alpha \alpha }$ we infer ${\textbf {e}}^\top _\alpha E_{\alpha \alpha } =0$. Similarly, by definition of $C_\alpha$ and $A_{\beta \alpha }$ we have that

$$\begin{aligned} {\textbf {e}}_\alpha ^\top C_\alpha = \sum _{\beta \in X \setminus \{\alpha \}} {\textbf {e}}^\top _\beta A_{\beta \alpha }. \end{aligned}$$

As a consequence of these observations we deduce that

$$\begin{aligned} \frac{d}{dt} N_\alpha (t)&=- \sum _{\beta \in X \setminus \{\alpha \}} {\textbf {e}}^\top _\beta A_{ \beta \alpha } n_\alpha (t) + \sum _{\beta \in X \setminus \{\alpha \} } {\textbf {e}}^\top _\alpha A_{ \alpha \beta } n_\beta (t) \\&= - \sum _{\beta \in X \setminus \{\alpha \}} \sum _{j \in \beta } \sum _{ i \in \alpha } (A_{ \beta \alpha })_{ji} (n_\alpha (t))_i + \sum _{i \in \alpha } \sum _{\beta \in X \setminus \{\alpha \} } \sum _{j \in \beta } (A_{ \alpha \beta })_{ij} (n_\beta (t))_j \\&= - \sum _{ i \in \alpha }\sum _{\beta \in X \setminus \{\alpha \}} \sum _{j \in \beta } I^{ij}_{\alpha \beta }(t) + \sum _{i \in \alpha } \sum _{\beta \in X \setminus \{\alpha \} } \sum _{j \in \beta } I^{ji}_{\beta \alpha }(t), \end{aligned}$$

with $I_{\alpha \beta }^{ij}(t)$ given by (29).

We now show that these fluxes satisfy (27) with corresponding kernels and forcing functions in (30) respectively (28). To this end, we note that due to (15) we have

$$\begin{aligned} n_\alpha (t)= e^{ t A_{\alpha \alpha }} n_\alpha ^0 + \sum _{\gamma \in X \setminus \{\alpha \} } \int _0^t e^{ (t-s)A_{\alpha \alpha } } A_{\alpha \gamma } n_\gamma (s) ds . \end{aligned}$$

Writing the *i*-th component of the above expression and multiplying by $\lambda _{ij}$ yields

$$\begin{aligned} I_{\alpha \beta }^{ij}(t)&= \lambda _{i j }( n_\alpha (t))_i \\&= \lambda _{i j } \sum _{r \in \alpha } \left( e^{t A_{\alpha \alpha } } \right) _{ir} (n_\alpha ^0)_r \\&\quad + \lambda _{i j } \sum _{r \in \alpha } \sum _{\gamma \in X \setminus \{\alpha \} } \sum _{ m \in \gamma } \int _0^t \left( e^{(t-s) A_{\alpha \alpha } }\right) _{ir}\left( A_{\alpha \gamma } \right) _{rm} (n_\gamma (s))_m ds \\&= \overline{I}_{\alpha \beta }^{ij} (t) + \sum _{r \in \alpha } \sum _{\gamma \in X \setminus \{\alpha \} } \sum _{ m \in \gamma } \int _0^t \Psi _{\alpha \beta }(t-s;r,i,j) \lambda _{ mr } (n_\gamma (s))_m ds \\&= \overline{I}_{\alpha \beta }^{ij} (t) + \sum _{r \in \alpha }\sum _{\gamma \in X \setminus \{\alpha \} } \sum _{ m \in \gamma } \int _0^t \Psi _{\alpha \beta }(t-s;r,i,j) I^{mr}_{\gamma \alpha }(s) ds \end{aligned}$$

This concludes the proof. $\square$

With this we can give the proof of Theorem 2.4.

#### Proof of Theorem 2.4

According to Lemma 2.5 the function $\{N_\alpha \}$ solves (26) with fluxes satisfying (29). Thus defining the fluxes $\{S_\alpha \}$, $\{J_\alpha \}$ by

31 $$\begin{aligned} (S_\alpha )_i = \sum _{\beta \in X \setminus \{\alpha \} } \sum _{j \in \beta } I_{\beta \alpha }^{ji}, \quad (J_\alpha )_i = \sum _{\beta \in X \setminus \{\alpha \} } \sum _{j \in \beta } I_{\alpha \beta }^{ij}. \end{aligned}$$

we obtain (16). Note that the formula in (25) is a consequence of (29) and (31). Moreover, from (27) we obtain

32 $$\begin{aligned} I_{ \alpha \beta }^{ij} (t)=\overline{I}_{\alpha \beta }^{ij} (t) + \sum _{ r \in \alpha } \int _0^t \Psi _{\alpha \beta }(t-s; r, i, j ) (S_\alpha (s))_r ds. \end{aligned}$$

Consequently, summing over $\beta \in X \setminus \{\alpha \}$ and over $j \in \beta$ yields that $\{ J_\alpha \}$ satisfies Eq. (18) with kernels and forcing functions given by

$$\begin{aligned} (K_{\alpha }(t))_{ir} = \sum _{\beta \in X \setminus \{\alpha \} } \sum _{j \in \beta } \Psi _{\alpha \beta }(t; r, i, j ), \quad (J_\alpha ^0(t))_i = \sum _{\beta \in X \setminus \{\alpha \} } \sum _{j \in \beta } \overline{I}_{\alpha \beta }^{ij} (t). \end{aligned}$$

Thus, (23) and (24) follow from the formula for the functions $\Psi _{\alpha \beta }$, cf. (30). We can argue similarly to deduce that $\{ S_\beta \}$ satisfy Eq. (17) by summing over $\alpha \in X$ and $i \in \alpha$ in Eq. (27) and defining

$$\begin{aligned} (G_{\alpha \beta }(t))_{jr} = \sum _{ i \in \alpha } \Psi _{\alpha \beta }(t; r, i, j ), \quad (S_\beta ^0(t))_i = \sum _{\alpha \in X \setminus \{\beta \} } \sum _{i \in \alpha } \overline{I}_{\alpha \beta }^{ij} (t) \end{aligned}$$

The two Eqs. (22) and (24) are, therefore, a consequence of (30). $\square$

#### Remark 2.2

The kernels given by (22) and (23) satisfy both the conditions in Lemma 2.2 and 2.3. Indeed, we have with for any $T\ge 0$

$$\begin{aligned} \int _0^T [{\textbf {e}}_\alpha ^\top K_\alpha (r)]_j\, dr&= \sum _{i \in \alpha }\sum _{\beta \in X\setminus \{\alpha \}}\sum _{k\in \beta } \int _0^T (A_{\beta \alpha })_{ki}\left( e^{tA_{\alpha \alpha }} \right) _{ij} \, dr \\&= \sum _{i \in \alpha }\int _0^T (C_{\alpha })_{ii}\left( e^{tA_{\alpha \alpha }} \right) _{ij} \, dr. \end{aligned}$$

Recall the definition of $C_\alpha$ in Sect. 2.1. Using $A_{\alpha \alpha }= E_{\alpha \alpha }-C_\alpha$ we obtain

$$\begin{aligned} \int _0^T {\textbf {e}}_\alpha ^\top K_\alpha (r)\, dr = {\textbf {e}}_\alpha ^\top \left( I - e^{TA_{\alpha \alpha }} \right) + {\textbf {e}}_\alpha ^\top \left( \int _0^TE_{\alpha \alpha }e^{tA_{\alpha \alpha }}\, dt \right) = {\textbf {e}}_\alpha ^\top \left( I - e^{TA_{\alpha \alpha }} \right) , \end{aligned}$$

since ${\textbf {e}}_\alpha ^\top E_{\alpha \alpha }=0$. Since the semigroup $e^{tA_{\alpha \alpha }}$ induced by $A_{\alpha \alpha }$ is positivity preserving we obtain the second inequality in (20). For the first in (20) we use (24) and the previous reasoning

$$\begin{aligned} \int _0^T{\textbf {e}}_\alpha ^\top K_\alpha (t)n^0_\alpha \, dt = {\textbf {e}}_\alpha ^\top \left( I - e^{TA_{\alpha \alpha }} \right) n^0_\alpha \le {\textbf {e}}_\alpha ^\top n^0_\alpha = N^0_\alpha . \end{aligned}$$

Finally, both conditions in (21) follow from (22) and (23). More precisely, we have for all $\alpha \in X$ and $k\in \alpha$

$$\begin{aligned}&\sum _{\beta \in X\setminus \{\alpha \}} [{\textbf {e}}_\beta ^\top G_{\alpha \beta }(t) ]_k- [{\textbf {e}}_\alpha ^\top K_\alpha (t)]_k \\&\qquad =\sum _{\beta \in X\setminus \{\alpha \}} \sum _{j \in \beta }\sum _{i\in \alpha } (A_{\beta \alpha })_{ji} \left( e^{tA_{\alpha \alpha }} \right) _{ik} - \sum _{\beta \in X\setminus \{\alpha \}} \sum _{j \in \beta }\sum _{i\in \alpha } (A_{\beta \alpha })_{ji} \left( e^{tA_{\alpha \alpha }} \right) _{ik} =0. \end{aligned}$$

We conclude this section with the special case of system of ODEs (10) with graph structure $(\Omega ,\mathcal {E})$ that is decomposed into compartments with at most one entrance point (see Definition 2.1). Then the system of Eqs. (16)–(18) can be reduced to (2)–(4).

#### Lemma 2.6

Consider $n(t) \in {\mathbb {R}}_+^{|\Omega |}$ solution to (14) and $N_\alpha$ defined in (12). Furthermore, assume that for all $\alpha \in X$ there is at most one entrance point in $(\Omega ,\mathcal {E})$, denoted by $i_\alpha$ if it exists. Then the following holds.

(i) For all $\alpha \in X$ we have $(S_\alpha ^0)_j=(S_\alpha )_j=0$ for all $j\ne i_\alpha$.
(ii) The functions $\{B_\alpha \}=\{(S_\alpha )_{i_\alpha }\}$, $\{D_\alpha \} = \{{\textbf {e}}_\alpha ^\top J_\alpha \}$ satisfy (3)–(4) with kernels and forcing functions
  $$\begin{aligned} k_\alpha&:= {\left\{ \begin{array}{ll} \sum _{j\in \alpha }(K_\alpha )_{j i_\alpha } & \text {if } i_\alpha \in \alpha \text { exists,} \\ 0 & \text {otherwise,} \end{array}\right. } \end{aligned}$$
  $$\begin{aligned} \Phi _{\beta \alpha } := {\left\{ \begin{array}{ll} (G_{ \beta \alpha } )_{i_\alpha i_\beta } & \text {if } i_\alpha \in \alpha ,\, i_\beta \in \beta \text { exist,} \\ 0 & \text {otherwise,} \end{array}\right. } \end{aligned}$$
  $$\begin{aligned} B_\alpha ^0&: = {\left\{ \begin{array}{ll} ( S^0_\alpha )_{i _\alpha } & \text {if } i_\alpha \in \alpha \text { exists,} \\ 0 & \text {otherwise,} \end{array}\right. } \qquad D_\alpha ^0 := {\textbf {e}}_\alpha ^\top J^0_\alpha . \end{aligned}$$
(iii) Finally, $\{N_\alpha \}$ satisfies (2) with fluxes $\{B_\alpha ,D_\alpha \}$.

#### Proof

We prove each claim separately.

*Claim (i).* Let us note that for $\alpha ,\, \beta \in X$ and $j\ne i_\alpha$, $j\in \beta$ we have $(A_{\alpha \beta })_{j\ell }= \lambda _{\ell j} =0$ for all $\ell \in \beta$. In particular, by (22) we have $(G_{\beta \alpha })_{jk} \equiv 0$ for all $k\in \beta$. Hence, (24) yields (i).

*Claim (ii).* By Theorem 2.4 the fluxes ${S_\alpha }$ solve the system (17). The $i_\alpha$-th component, if it exists, yields (3) for $\Phi _{\beta \alpha }$ as in (ii) due to (i).

Furthermore, $\{J_\alpha \}$ solve (18). Thus, (i) and summing over $j\in \alpha$ yields (4) with kernels $\Phi _{\alpha }$ as in (ii).

*Claim (iii).* Finally, we conclude (iii) from (16) and $B_\alpha = {\textbf {e}}_\alpha ^\top S_\alpha = (S_\alpha )_{i_\alpha }$ if $i_\alpha$ exists, $B_\alpha =0$ otherwise. $\square$

#### Remark 2.3

Let us recall that the kernels $\{\Phi _{ \alpha \beta }\}_{\alpha ,\beta \in X}$ in (2)–(4) are assumed to satisfy

33 $$\begin{aligned} \sum _{\beta \in X\setminus \{\alpha \}} \int _0^\infty \Phi _{ \alpha \beta }(t)\, dt =1. \end{aligned}$$

if the compartment $\alpha$ is not a sink, i.e., if there are connections to any other compartment $\beta \ne \alpha$. In the latter case the kernels are zero $\Phi _{\alpha \beta }\equiv 0$ for all $\beta \ne \alpha$.

However, even if $\Phi _{\alpha \beta }\ne 0$ a further assumption on the compartment $\alpha$ is necessary to obtain (33). Observe that from (ii) in Lemma 2.6 and form (22) it follows that

$$\begin{aligned} \Phi _{\alpha \beta }(t) =\left( A_{\beta \alpha }e^{t A_{\alpha \alpha }} \right) _{i_\beta , i_\alpha }. \end{aligned}$$

Thus, $\Phi _{\alpha \beta }\ne 0$ implies that there is a path in $(\Omega ,\mathcal {E})$ from $i_\alpha$ to $i_\beta$. However, in $\alpha$ there could be a state $\Delta \in \alpha$ that has no path to any entrance point $i_\beta$, $\beta \ne \alpha$, but there is a path from $i_\alpha$ to $\Delta$. Hence, there is a sink inside the compartment $\alpha$. In this case, (33) fails. The reason is that $\lim _{t\rightarrow \infty } e^{tA_{\alpha \alpha }}e_{i_\alpha }\ne 0$. In fact, the left hand side in (33) can be reformulated as follows. Note that $(A_{\beta \alpha })_{j,i}=0$ for $j\ne i_\beta$ and any $i\in \alpha$, since $i_\beta$ is the only entrance point, cf. Definition 2.1. We conclude from this and the conservation property $\sum _{ \beta \in X \setminus \{\alpha \} } {\textbf {e}}_{|\beta |}^\top A_{\beta \alpha } = -{\textbf {e}}_{|\alpha |}^\top A_{\alpha \alpha }$ that

$$\begin{aligned} \sum _{\beta \in X\setminus \{\alpha \}} \int _0^\infty \Phi _{ \alpha \beta }(t)\, dt = -{\textbf {e}}_{|\alpha |}^\top \int _0^\infty A_{\alpha \alpha }e^{tA_{\alpha \alpha }} e_{i_\alpha } \, dt = 1-\lim _{t\rightarrow \infty } {\textbf {e}}_{|\alpha |}^\top e^{tA_{\alpha \alpha }} e_{i_\alpha }. \end{aligned}$$

Thus, we need necessarily $\lim _{t\rightarrow \infty } {\textbf {e}}_{|\alpha |}^\top e^{tA_{\alpha \alpha }} e_{i_\alpha }=0$ to ensure (33). This is satisfied if any state $j\in \alpha$ that has a path from $i_\alpha$ to *j* also has a path to some entrance point $i_\beta$, $\beta \ne \alpha$.

## Approximation of measures by means of response functions

In this section, we are concerned with approximation results for the kernels $\{\Phi _{ \alpha \beta }\}_{\alpha ,\beta \in X}$ appearing in (3). We restrict ourselves to the case that each compartment has at most one entrance point. Accordingly, the RFEs have the form (2)–(4). In Sect. 3.1 we prove that the set of the response functions obtained form ODEs of the form (10) is dense in the space of probability measures. In Sect. 3.2, we prove that if we assume that (10) satisfies the detailed balance condition, then the response functions must be completely monotone functions.

### Density of the response functions obtained from ODEs in the space of probability measures

Recall that in Sect. 2 we proved that a system of ODEs of the form (10) can be reduced to the system (2)–(4) by decomposing the state space into compartments with at most one entrance point, cf. Theorem 2.4 and Lemma 2.6. Furthermore, the corresponding kernels $\{\Psi _{ \alpha \beta }\}_{\alpha ,\beta \in X}$ have the form

34 $$\begin{aligned} \Psi _{\alpha \beta }(t) =\left( A_{\beta \alpha }e^{t A_{\alpha \alpha }} \right) _{i_\beta , i_\alpha } = \sum _{j \in \alpha } \left( A_{\beta \alpha } \right) _{i_\beta , j}\left( e^{t A_{\alpha \alpha }} \right) _{j,i_\alpha }= \sum _{j \in \alpha } \lambda _{j, i_\beta }\left( e^{t A_{\alpha \alpha }} \right) _{j,i_\alpha } , \end{aligned}$$

where $i_\alpha \in \alpha ,\, i_\beta \in \beta$ are the entrance points in $\alpha$ and $\beta$, if they exist, $\Psi _{\alpha \beta }\equiv 0$ otherwise.

We now show that for any set of integral kernels $\{\Phi _{ \beta \alpha }\}_{\beta ,\alpha \in X}$ satisfying (7) it is possible to find a sequence of kernels $\{\Psi _{\alpha \beta }\}$ as in (34) that is arbitrarily close (in a suitable sense) to the kernel $\{\Phi _{ \beta \alpha }\}_{\beta ,\alpha \in X}$. In other words, we prove that for any system of the form (2)–(4) we can find an approximating sequence of systems of ODEs of the form (10).

In order to prove this we need to specify

(i) a state space $\Omega '$, on which the approximating ODEs will be defined;
(ii) the jump rates between the states, i.e. a matrix $A\in \mathbb {R}^{|\Omega '|\times |\Omega '|}$ yielding an ODE system (10). This defines a directed graph $(\Omega ',\mathcal {E}')$;
(iii) a decomposition $X'\subset 2^{\Omega '}$ into compartments.

Since the kernels $\{\Phi _{ \alpha \beta }\}_{\beta ,\alpha \in X}$ are labeled by the set *X*, the decomposition $X'$ needs to be identified with *X*. More precisely, we require the existence of a relabeling, i.e. there is a bijective mapping $\iota :X\rightarrow X'$ which associates to each compartment $\alpha \in X$ a compartment $\iota (\alpha )=\alpha '\in X'$. Then, we apply the procedure in Sect. 2 to $(\Omega ',\mathcal {E}')$ and the decomposition $X'$ which yields kernels $\{\Psi _{\beta '\alpha '}\}_{\beta ',\alpha '\in X'}$. Choosing $(\Omega ',\mathcal {E}')$ and $X'$ appropriately, these kernels yield an approximation to $\{\Phi _{ \alpha \beta }\}_{\beta ,\alpha \in X}$.

The approximation result holds in terms of the weak topology of measures. Recall that we denote by $d_w$ a metric inducing weak convergence, see Sect. 1.1. Let us note that in the precise statement below we assume that the response functions are merely finite, non-negative measures $\Phi _{\alpha \beta }\in \mathcal {M}_{+,b}(\mathbb {R}_+)$ rather than measurable functions.

#### Theorem 3.1

Consider a family of finite, non-negative measure kernels $\{\Phi _{ \alpha \beta }\}_{\beta ,\alpha \in X}$ with $|X|<\infty$ satisfying (7) or (8). Let $\varepsilon >0$ be arbitrary. Then, we can find a finite state space $\Omega '$, a decomposition $X'\subset 2^{\Omega '}$ and a matrix $A\in \mathbb {R}^{|\Omega '|\times |\Omega '|}$ with the following properties.

(i) The matrix *A* satisfies $A_{ij}\ge 0$ for all $i\ne j$, $i,\, j\in \Omega '$ and ${\textbf {e}}_{|\Omega '|}^\top A=0$.
(ii) There is a bijection $\iota : X\rightarrow X'$.
(iii) The partition $X'$ decomposes $(\Omega ';\mathcal {E}')$ into compartments with at most one entrance point, denoted by $i_{\alpha }\in \alpha$, $\alpha \in X'$ if it exists, see Definition 2.1.
(iv) The kernels given by
  35 $$\begin{aligned} \Psi _{ \alpha \beta }(t) = \sum _{j \in \alpha } \left( A_{\beta \alpha } \right) _{i_\beta , j}\left( e^{t A_{\alpha \alpha }} \right) _{j,i_\alpha } \end{aligned}$$
  if $i_{\alpha }, \, \omega _{\alpha ,\beta }\in \alpha$ exist, $\Psi _{ \alpha \beta }\equiv 0$ otherwise are such that
  $$\begin{aligned} \int _0^\infty \Psi _{ \alpha \beta }(t)\, dt = \Phi _{ \alpha \beta }(\mathbb {R}_+) =: p_{\alpha \beta }, \end{aligned}$$
  $$\begin{aligned} \sum _{\alpha ,\beta \in X, \, p_{\alpha \beta }>0} d_w \left( \frac{1}{p_{\alpha \beta }}\Psi _{ \iota (\alpha ),\iota (\beta ) }(t)\, dt, \, \frac{1}{p_{\alpha \beta }}\Phi _{ \alpha \beta }\right) \le \varepsilon . \end{aligned}$$

Furthermore, let us mention that the above result is reminiscent of the so-called *phase method* in queuing theory, see e.g. (Asmussen (2003), Section III.4), allowing to approximate any probability measure on $(0,\infty )$ by a combination of exponentially distributed times. For the proof of Theorem 3.1 we make use of this construction which contains probabilistic arguments.

#### Proof of Theorem 3.1

We split the proof into two steps. In the first step we recall a standard result from queuing theory which allows to approximate any probability distribution on $\mathbb {R}_+$. Furthermore, we show how to obtain such approximations from ODE models of the form (10) for a certain choice of matrices *A*. In the context of the above theorem, this allows to approximate one given response function by an ODE model. This serves as a basic building block for the next step. In Step 2 we then show how to construct the whole state space $\Omega '$ with a partition $X'$ as well as the matrix *A* that allows to find an approximation for all response functions $\{\Phi _{\alpha \beta }\}$ simultaneously.

*Step 1.* Let us recall the following result from (Asmussen (2003), Theorem 4.2): any probability measure $\Phi$ on $(0,\infty )$ can be approximated in the weak topology by distributions *F* with density

36 $$\begin{aligned} f(t):=\sum _j^L q_j \gamma _{M,m_j}(t), \quad \gamma _{M,m}(t) = \dfrac{M^{m+1}}{m!} t^m e^{-M t}, \quad t>0 \end{aligned}$$

for some $$ L\in \mathbb {N}$$, $$ q_j>0$$, $$ \sum _j q_j=1 $$ and some $$ M\in \mathbb {N}$$, $$ m_j\in \mathbb {N}$$. Here, $$ \gamma _{M,m} $$ is the density of the Gamma distribution appearing as an *m*-fold convolution of the exponential distribution with rate $$ M\in \mathbb {N}$$. Thus, for $$ \varepsilon >0 $$ we can find corresponding parameters such that $$ d_w(F,\Phi )<\varepsilon $$ with the measure $$ F= f(t)\, dt $$.

**Fig. 2:**
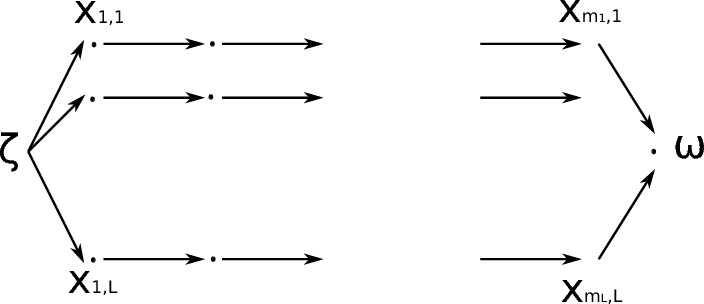
Graph structure corresponding to approximation in Step 1

We now construct an ODE system of the form (10) and relate *f*(*t*) in (36) with the response function corresponding to the ODEs system. We start by describing the structure of the graph. Consider one starting node $$ \zeta $$ and *L* chains with $$ m_j$$ nodes (that we denote with $$x_{n j } $$ with $$1 \le j \le L $$ and $$1 \le n \le m_j $$) bifurcating from $$ \zeta $$. Furthermore, all chains have $$ \omega $$ as final node, see Fig. 2.

We assume that the rate to jump from $$\zeta $$ to $$x_{1 j } $$ is equal to $$q_j M$$ while the rate to jump from $$x_{n-1, j}$$ to $$x_{n, j}$$ is equal to *M*. Finally the rate to go from $$x_{m_{j}}$$ to $$\omega $$ is also equal to *M*. This defines the ODE system

37 $$\begin{aligned} \begin{aligned} \frac{d \zeta }{dt}&= - M \zeta , \quad \zeta (0)=1 \\ \frac{d x_{1,j }}{dt }&= q_j M \zeta - M x_{1j}, \quad x_{1, j }(0)=0, \quad \text { for } 1 \le j \le L \\ \frac{d x_{n, j }}{dt }&= M x_{n-1, j} - M x_{n, j}, \quad x_{n, j }(0)=0, \quad \text { for } 1 \le j \le L \text { and } 2 \le n \le m_j \end{aligned} \end{aligned}$$

which can be written also as $\frac{dx}{dt}= A x$ where $x=(\zeta , x_{1, 1}, \dots x_{m_L, L })$ and where the matrix *A* is such that $A_{ij}\ge 0$ for $i\ne j$ and ${\textbf {e}}_n^\top A=0$.

We now prove that $M\sum _{j=1}^L x_{m_{j}-1, j } (t) = f(t)$. Indeed

38 $$\begin{aligned} \hat{f}(z)= \sum _{j =1 }^L q_j \widehat{\gamma _{M , m_j} }(z) = \sum _{j =1 }^L \frac{ q_j M^{m_j +1}}{( z + M )^{m_j+1}} = M \sum _{j=1 }^L \widehat{x_{m_j, j }}(z) \end{aligned}$$

where the last equality has been obtained solving (37) via Laplace transforms. Equality (38) implies that $M\sum _{j \in L} x_{m_{j}, j } (t) = f(t)$. Notice that if $\alpha =\{\zeta , x_{1,1}, \dots x_{m_{L}, L } \}$ and $\beta =\{ \omega \}$ are two compartments, then *f*(*t*) is the response function $\Phi _{\alpha \beta }$ in Lemma 2.6.

*Step 2.* We now use Step 1 to construct the state space $\Omega '$, the matrix *A* and the partition $X'$. To this end, we construct as in Step 1 probability measures $F_{\alpha \beta }$, for each pair of compartments, with densities $f_{\alpha \beta }$ of the form (36) that satisfy

39 $$\begin{aligned} \sum _{\alpha , \beta \in X, \, p_{\alpha \beta }>0} d_w \left( F_{\alpha \beta }, \dfrac{1}{p_{\alpha \beta }}\Phi _{ \alpha \beta }\right) \le \varepsilon . \end{aligned}$$

Observe that $\frac{1}{p_{\alpha \beta }}\Phi _{ \alpha \beta }$ is a probability measure.

We now construct the ODE system and its corresponding graph. First, let $(i_\alpha )_{\alpha \in X}$ be states representing the entrance points of the compartments to be constructed. Next, we fix one compartment $\alpha \in X$ and specify the states and rates inside the compartment $\alpha '=\iota (\alpha )\subset \Omega '$. For any $\beta \ne \alpha$ with $p_{\alpha \beta }>0$ introduce a state $\zeta _{\alpha \beta }$. The rate to jump from $i_\alpha$ to $\zeta _{\alpha \beta }$ is given by $Mp_{\alpha \beta }$.

**Fig. 3:**
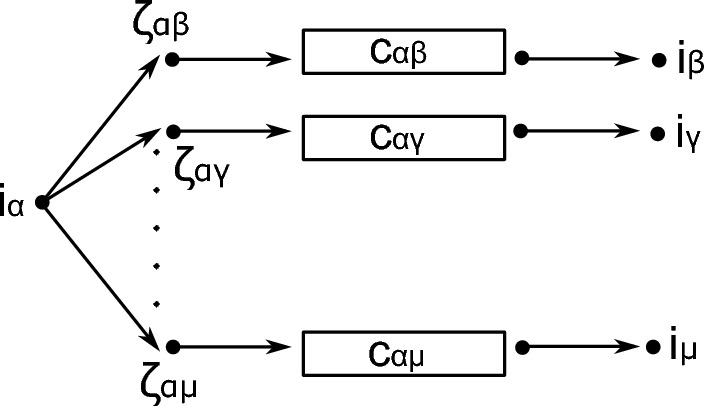
Graph structure in compartments in Step 2. Here with $\{C_{\alpha \beta } \}_{\alpha \ne \beta }$ we denote the graphs that connect $\zeta =\zeta _{\alpha \beta }$ to $\omega =i_\beta$ as in Fig. 2

Now we add to the state $\zeta _{\alpha \beta }$ the graph in Step 1, see Fig. 3, with end point $\omega :=i_\beta$.

We can repeat this construction for any compartment and we obtain the state space $\Omega '$, the partition $X'$, the relabeling $\iota : X\rightarrow X'$ and the matrix *A*. By definition *A* satisfies statement (i) in the theorem. Also statements (ii) and (iii) are satisfied.

Finally, concerning (iv) we use Step 1 to deduce that for every $\alpha \ne \beta$ the approximation (39) holds for the measures $F_{\alpha \beta }$ with densities given by

$$\begin{aligned} f_{\alpha \beta }(t) = \sum _{j \in \alpha } \left( A_{\beta \alpha } \right) _{i_\beta , j}\left( e^{t A_{\alpha \alpha }} \right) _{j,i_\alpha }=\Psi _{\alpha \beta }(t). \end{aligned}$$

$$\square $$

### Response functions corresponding to ODEs satisfying the detailed balance condition

In this section we demonstrate that a particular structure of the biochemistry of the system imposes some restriction on the form of the response functions. Specifically we consider here systems satisfying the detailed balance condition.

We say that the ODE model (10) satisfies the detailed balance condition if there is $\mu \in \mathbb {R}_+^{|\Omega |}$, $\mu _j>0$, $\sum _{i\in \Omega }\mu _i=1$ such that $A\mu =0$ and $A_{ij}\mu _j=A_{ji}\mu _i$ for all $i,j\in \Omega$. The latter condition is equivalent to the fact that $M^{-1}AM$ is a symmetric matrix, where

$$\begin{aligned} M:=\operatorname {diag}\left( \{\sqrt{\mu _j}\}_{j\in \Omega } \right) . \end{aligned}$$

Let us mention that the vector $\mu$ is usually referred to as the equilibrium distribution of the ODE system. We will prove, see Theorem 3.2, that the corresponding response functions are completely monotone. We recall that a completely monotone function $f:\mathbb {R}_+\rightarrow \mathbb {R}$ is continuous on $\mathbb {R}_+$ and satisfies $(-1)^nf^{(n)}(t)\ge 0$ for all $n\in \mathbb {N}_0$, $t>0$. An example of completely monotone function is the exponential function $e^{- \lambda t}$ with $\lambda \ge 0$ or the function 1/*t* for $t>0$. It is well-known that completely monotone functions are exactly the Laplace transforms of non-negative finite Borel measures on $\mathbb {R}_+$. See for instance (Feller 1967, Chapter XIII).

We have the following result.

#### Theorem 3.2

Let (10) with $A\in \mathbb {R}^{|\Omega |\times |\Omega |}$ satisfy detailed balance for $\mu \in \mathbb {R}_+^{|\Omega |}$. Let *X* be a decomposition such that any compartment has at most one entrance point in $(\Omega ,\mathcal {E})$. Then, we have for all $\alpha ,\, \beta \in X$, $\alpha \ne \beta$ and some $\kappa _j,\, \nu _j\ge 0$

$$\begin{aligned} \Phi _{ \beta \alpha }(t) = \lambda _{i_\beta ,i_\alpha } \sum _{j \in \beta } \kappa _j^2 e^{-\nu _j t}, \quad \sum _{j \in \beta } \kappa _j^2=1, \end{aligned}$$

whenever the entrance points $i_\alpha \in \alpha$, $i_\beta \in \beta$ exists, otherwise $\Phi _{ \beta \alpha }\equiv 0$. In particular, $\Phi _{ \beta \alpha }$ is completely monotone.

Theorem 3.2 implies that not all the sets of kernels $\{\Phi _{ \beta \alpha }\}$ can be approximated by those appearing in an ODE model (10) with detailed balance. This is in contrast with Theorem 3.1 and implies a very strong restriction on the integral kernels that can be approximated by models satisfying the detailed balance condition.

#### Proof of Theorem 3.2

Let $\alpha ,\, \beta \in X$ with entrance points $i_\alpha ,\, i_\beta$, respectively. By Theorem 2.4 and Lemma 2.6 we have

$$\begin{aligned} \Phi _{\beta \alpha }(t)= \left( A_{\alpha \beta } e^{A_{\beta \beta }t}\right) _{i_\alpha ,i_\beta } = \lambda _{i_\beta ,i_\alpha } \left( e^{A_{\beta \beta }t}\right) _{i_\beta ,i_\beta }. \end{aligned}$$

Here, we used that $A_{\beta \alpha }$ has only one non-zero element due to the detailed balance condition and the assumption that there is at most one entrance point in $\alpha , \, \beta$. Let us define $M^\beta := \operatorname {diag}(\{\sqrt{\mu _j}\}_{j\in \beta })$. Using the decomposition $A_{\beta \beta }=E_{\beta \beta }-C_{\beta }$ as in Sect. 2.1 we obtain that $D_{\beta \beta }:=(M^\beta )^{-1}A_{\beta \beta }M^\beta$ is symmetric because the matrix *A* satisfies the detailed balance condition and therefore the blocks $A_{\beta \beta }$ also satisfy the detailed balance condition. Recall that $E_{\beta \beta }$ defines a conservative ODE system, while $-C_{\beta }$ is an additional loss term in $A_{\beta \beta }=E_{\beta \beta }-C_{\beta }$. Thus, the eigenvalues $\{\nu _j\}_{j\in \beta }$ of $D_{\beta \beta }$ are non-positive. Using an orthonormal basis of eigenvectors we deduce that there is an orthogonal matrix *Q* such that

$$\begin{aligned} \Phi _{\beta \alpha }(t) = \lambda _{i_\beta ,i_\alpha } \left( Q\operatorname {diag}\left( \{e^{-\nu _jt}\}_{j\in \beta } \right) Q^\top \right) _{i_\beta ,i_\beta } = \lambda _{i_\beta ,i_\alpha } \sum _{j \in \beta } Q_{j,i_\beta }^2e^{-\nu _jt}. \end{aligned}$$

Since *Q* is orthogonal we have $\sum _{j \in \beta }Q_{j,i_\beta }^2 =1$. Defining $\kappa _j:=Q_{j,i_\beta }$ concludes the proof. $\square$

## Characterization of response functions yielding Markovian dynamics

As anticipated in the introduction, one important property that is lost when reformulating the system of ODEs (14) using the response function formalism (16)–(18), is the Markovianity of the evolution of the number of individuals $\{N_\alpha \}$.

In this section, we give a precise definition of Markovianity and characterize the integral kernels yielding a Markovian dynamics. To this end, we restrict ourselves to decompositions *X* of the state $\Omega$ into compartments with at most one entrance point. In other words, we restrict our attention to RFEs of the form (2)–(4) with forcing function satisfying particular conditions (see (42)).

As we will see, kernels inducing a Markovian dynamics are exactly given by exponentials, and they correspond to Markov jump processes involving exponentially distributed waiting times, see Theorem 4.2 below. Similar results, for some type of non-linear structured population models can be found in Diekmann et al. (2020). Here we present a simple proof for the linear renewal equations considered in this paper.

### Definition 4.1

(Markovianity) We say that the evolution induced by (2)–(4) is Markovian if and only if any solution *N* of Eq. (2) can be written in the form

40 $$\begin{aligned} N (t)= e^{t A} N^0 \end{aligned}$$

for all $N^0\in \mathbb {R}^{|X|}$. Furthermore, the matrix $A \in {\mathbb {R}}^{|X|\times |X|}$ is independent of $N^0 \in {\mathbb {R}}_+^{|X|}$, satisfying $A_{ii} = - \sum _{i \ne k } A_{ik}$ for every $i \in \{1, \dots , |X|\}$.

Since we apply the Laplace transform in the proof of Theorem 4.2 below, the following lemma is useful.

### Lemma 4.1

Assume that each compartment in $$\alpha \in X$$ has at most one entrance point. Let $$z_0> 0 $$ such that $$ \{ k_\alpha \},\, \{ \Phi _{\alpha \beta } \}, \, \{ D_\alpha ^0 \},\{ B_\alpha ^0 \} \in L^1({\mathbb {R}}_+, e^{- z_0 t} dt )$$ for every $$\alpha ,\, \beta \in X$$.

Then, there exists a unique solution $\{N_\alpha , B_\alpha , D_\alpha \}$ to (2)–(4) such that for every $\alpha \in X$ it holds that $B_\alpha ,\, D_\alpha \in L^1({\mathbb {R}}_+, e^{- x_0 t} dt)$ for some $x_0 > z_0$ and $N_\alpha \in W^{1,1}({\mathbb {R}}_+, {\mathbb {R}}_+ )$ satisfies

41 $$\begin{aligned} \int _0^\infty N_\alpha (t) e^{- x_0 t } dt < \infty . \end{aligned}$$

### Proof

We refer to (Gripenberg et al. (1990), Section 3.3) for the existence of $$B_\alpha \in L^1({\mathbb {R}}_+, e^{- x_0 t} dt)$$ for some $$x_0 > z_0 $$. Thanks to the assumptions on $$k_\alpha $$ and $$D^0_\alpha $$ we deduce that $$D_\alpha \in L^1({\mathbb {R}}_+, e^{- x_0 t} dt)$$. Hence (41) follows by Gronwall’s Theorem. $$\square $$

### Theorem 4.2

(Markovianity for RFEs with at most one entrance point) Under the conditions of Lemma 4.1, assume moreover that $\{ k_\alpha \}$, $\{ \Phi _{\alpha \beta } \}$ satisfy (5) and that for every $\alpha \in X$ either equality (7) or (8) holds. Let $\{N_\alpha , B_\alpha , D_\alpha \}$ be the solution to (2)–(4) with

42 $$\begin{aligned} D_\alpha ^0 (t)=k_\alpha (t) N^0_\alpha , \quad B_\alpha ^0(t)= \sum _{\beta \in X \setminus \{ \alpha \} } \Phi _{\alpha \beta }(t) N_\beta ^0 \end{aligned}$$

for every $t \ge 0$ and $N^0\in {\mathbb {R}}^{|X|}$. Then, the evolution of $\{N_\alpha \}$ is Markovian, in the sense of Definition 4.1, if and only if for all $t \ge 0$

43 $$\begin{aligned} { \Phi _{\alpha \beta } (t)= \lambda _{ \alpha \beta } e^{- t \sum _{\gamma \in X \setminus \{ \alpha \} } \lambda _{\alpha \gamma } }} \end{aligned}$$

for some $$\lambda _{\alpha \beta } \in {\mathbb {R}}_+ $$.

### Proof

*Step 1.* We first assume that the dynamics are Markovian and deduce (43). Let $\Delta :=\left\{ z \in {\mathbb {C}}: \Re z > x_0 \right\}$. We apply the Laplace transform to each term in (3)–(4) yielding

44 $$\begin{aligned} \hat{D}(z)&= \hat{D}^0(z) + Q(z) \hat{B} (z) \end{aligned}$$

45 $$\begin{aligned} \hat{B}(z)&= \hat{B}^0(z) + M(z) \hat{B} (z) \end{aligned}$$

for $z \in \Delta$. Here, $\hat{D}(z) \in {\mathbb {R}}_+^{|X|}$ is the vector whose $\alpha$-th element is the Laplace transform of $D_\alpha$ and we define $\hat{D}^0(z),\, \hat{B}^0(z),\, \hat{B}(z) \in {\mathbb {R}}_+^{|X|}$ analogously. The matrix *Q*(*z*) is diagonal with $(Q(z))_{\alpha \alpha }=\hat{k}_\alpha (z)$ and $(M(z))_{\alpha \beta }= \hat{\Phi }_{\beta \alpha }(z)$.

Similarly, we obtain from (2) that

46 $$\begin{aligned} z\hat{N} (z) = N^0+ \hat{B} (z) - \hat{D} (z), \end{aligned}$$

for $z \in \Delta$. Notice that $z \mapsto (I - M(z))^{-1}$ is a meromorphic map on $\Delta$. We denote by *P* the set of poles in $\Delta$. From Eq. (45) we deduce that for $z \in \Delta \setminus P$ it holds that

47 $$\begin{aligned} \hat{B}(z)=\left( I - M(z) \right) ^{-1}\hat{B}^0(z). \end{aligned}$$

Substituting this in (46) and using (44) we obtain

48 $$\begin{aligned} z \hat{N} (z) = N^0+ \left( I-Q(z) \right) \left( I - M(z) \right) ^{-1}\hat{B}^0(z)- \hat{D}^0(z) \end{aligned}$$

for $z \in \Delta \setminus P$. By assumption there exists a matrix *A* with $\frac{d N}{ dt} = AN$. Since *N* satisfies (41) the spectral radius of the matrix *A* is smaller or equal than $x_0$. Applying the Laplace transform yields

49 $$\begin{aligned} \hat{N}(z)= (z I- A )^{-1} N^0. \end{aligned}$$

for $z\in \Delta$. Thus, combining (48), (49) and $\hat{B}^0(z) = M(z) N^0$, $\hat{D}^0(z) = Q(z) N^0$ we obtain

$$\begin{aligned} A (zI - A)^{-1} N^0 = \left( I - Q(z) \right) \left( I - M(z) \right) ^{-1}M (z) N^0- Q(z) N^0 \end{aligned}$$

for $z\in \Delta \setminus P$. Since this holds for any $N^0\in \mathbb {R}^{|X|}$ this implies

$$\begin{aligned} A (z I - A)^{-1}&= \left( I - Q(z) \right) \left( I - M(z) \right) ^{-1}M (z) - Q(z) \\&= - I + \left( I - Q(z) \right) \left( I - M(z) \right) ^{-1}. \end{aligned}$$

After rearranging we obtain

50 $$\begin{aligned} z M(z) = z Q(z)+ A(I - Q(z)) \end{aligned}$$

for $z \in \Delta \setminus P$. Now recall that *Q*(*z*) is diagonal with $Q_{\alpha \alpha }(z)= \hat{k}_\alpha (z)$ and $(M(z))_{\alpha \alpha } =0$. Thus, (50) yields for $z\in \Delta \setminus P$

51 $$\begin{aligned} z\hat{k}_{ \alpha }(z) + \lambda _{\alpha \alpha }(1- \hat{k}_\alpha (z)) =0 \end{aligned}$$

where $\lambda _{\alpha \alpha }:= A_{\alpha \alpha }$. When instead $\alpha \ne \beta$ then

52 $$\begin{aligned} z \hat{\Phi }_{\alpha \beta }(z)= \lambda _{ \alpha \beta } (1- \hat{k}_\alpha (z) ) \end{aligned}$$

for $z\in \Delta \setminus P$, where $\lambda _{\alpha \beta }:= A_{\beta \alpha }$. Thus, (51) and (52) yield

53 $$\begin{aligned} z \hat{\Phi }_{\alpha \beta } (z)= \lambda _{ \alpha \beta } \left( 1+ \frac{\lambda _{\alpha \alpha }}{z- \lambda _{\alpha \alpha }} \right) = z\frac{\lambda _{\alpha \beta }}{z-\lambda _{\alpha \alpha }} \end{aligned}$$

for $z \in \Delta \setminus P$. We deduce that $\Phi _{\alpha \beta }(t)= \lambda _{ \alpha \beta } e^{t \lambda _{\alpha \alpha }}$ and that $k_{\alpha } (t)= - \lambda _{\alpha \alpha } e^{t \lambda _{\alpha \alpha }}$. Then, (43) follows from $\lambda _{\alpha \alpha } = - \sum _{\beta \in X \setminus \{\alpha \} } \lambda _{\alpha \beta }$.

*Step 2.* Assume now that $\Phi$ is given by (43). Then we have $\hat{\Phi }_{\alpha \beta } (z)= \lambda _{\alpha \beta }/(z-\lambda _{\alpha \alpha })$ and $\hat{k}_\alpha (z)=-\lambda _{\alpha \alpha }/(z-\lambda _{\alpha \alpha })$ for $z>0$, where $\lambda _{\alpha \alpha } = - \sum _{\beta \in X \setminus \{\alpha \} } \lambda _{\beta \alpha }$. Let us define the matrix *M*(*z*) via $M_{\alpha \alpha }(z)=0$, $M_{\alpha \beta }(z)=\hat{\Phi }_{\beta \alpha } (z)= \lambda _{\beta \alpha }/(z-\lambda _{\beta \beta })$ and the diagonal matrix *Q*(*z*) via $Q_{\alpha \alpha } (z) =\hat{k}_\alpha (z)= - \lambda _{\alpha \alpha }/(z-\lambda _{\alpha \alpha })$. As in Step 1, we apply the Laplace transform to (2)–(4) and use (47), yielding for $z>0$

$$\begin{aligned} z \hat{N}(z)&= N^0+(I-Q(z)) (I-M(z))^{-1} M(z) N^0 - Q(z) N^0 \\&= (I-Q(z))(1-M(z))^{-1}N^0. \end{aligned}$$

Thus, the matrix $$A(z):= z-z(I-M(z))(I-Q(z))^{-1} $$ satisfies $$\hat{N}(z) =( z I -A)^{-1} N^0$$. To conclude, notice that $$A_{\alpha \beta } (z)= \lambda _{\beta \alpha } $$ when $$\alpha \ne \beta $$ while $$A_{\alpha \alpha } (z)= \lambda _{\alpha \alpha } $$. In particular, *A* is independent of *z*. Thus, the requirements of Definition 4.1 are satisfied. $$\square $$

## Long-time behaviour of the solution of the classical RFEs

In this section we study the long-time behaviour of equations of the form (2)–(4). Once the asymptotic behaviour of *B*(*t*) is known, the one of the vector *D*(*t*) follows from the equality (4). Similarly the long-time behaviour of *N*, satisfying (2), can be obtained starting from the asymptotics of *B* and *D*.

To study the long-time behaviour of *B*(*t*) we will need to make an additional assumption on the kernels $\Phi$. More precisely we assume that the matrix $\Phi$ such that $(\Phi )_{\alpha \beta } = \Phi _{\beta \alpha }$ is irreducible. This corresponds to assuming that every compartment $\alpha$ can be reached by any other compartment $\beta$. In other words the graph having as vertexes the compartments must be strongly connected. This assumption guarantees the existence of an invariant measure, which is proven here to be stable. The irreducibility assumption of $\Phi$ corresponds to the irreducibility property which guarantees the existence of and convergence to an invariant measure for Markov chains (Feller (1967), Chapter VIII).

To analyse the long-time behaviour of (3) we will use Laplace transforms. In particular, we follow the approach in Diekmann et al. (2012). In order to apply Laplace transforms we make the same assumptions as in Theorem 4.2 on the forcing function $B^0$ and on the kernel $\Phi$. We also use the same notation as in the proof of Theorem 4.2 for $M(z), \Delta , P$. Namely $\Delta$ is the subset of the complex plain on which the Laplace transform is defined, $(M(z))_{\alpha \beta } = \hat{\Phi }_{\beta \alpha }(z)$ for $z \in \Delta$ and *P* is the set of the poles of the map $z \mapsto (I - M(z))^{-1}$, which is meromorphic on $\Delta$. For $z \in \Delta \setminus P$ we have, cf. (47),

54 $$\begin{aligned} \hat{B}(z)=\left( I - M(z) \right) ^{-1}\hat{B}^0(z). \end{aligned}$$

The first step for analysing the long-time behaviour of *B* consists in finding a solution (*r*, *v*) , with $r \in {\mathbb {R}}$ and $v \in {\mathbb {R}}_+^{|X|}$, to the non-linear eigenproblem

55 $$\begin{aligned} v= M(r) v. \end{aligned}$$

We call this eigenproblem non-linear to distinguish it from the classical eigenproblem of the form $Mv=r v$. In this case the non-linearity is due to the non-linear dependence of *M*(*r*) on *r*. Notice that the solutions *r* of Eq. (55) are the poles of the function $z \mapsto I-M(z)$.

### Lemma 5.1

Let the assumptions of Lemma 4.1 hold. Moreover, assume that for every $$z \in {\mathbb {R}}$$ the matrix *M*(*z*) is irreducible, i.e. for every $$i \ne j $$ there exists a $$k >0$$ such that $$(M(z)^k)_{ij} \ne 0$$. Then there exists a unique real eigen-couple $$(z, v)=(0, v_0 ) $$ satisfying Eq. (55) with $$v_0 \in {\mathbb {R}}_+^{|X|} $$.

### Proof

We recall that by the Perron–Frobenius theorem for irreducible matrices, see (Carl 2000), for every $$z \in {\mathbb {R}} $$ the spectral radius $$\rho (M(z))$$ of the matrix *M*(*z*) is a simple eigenvalue of *M*(*z*) . Moreover, the corresponding eigenvector $$v_r $$ has positive entries $$v_r \in {\mathbb {R}}_+^{|X|}$$.

By the definition of *M*(*z*) , ${\textbf {e}}_{|X|}^T$ is a left-eigenvector of *M*(0), hence the matrix *M*(0) has only one eigenvalue $\lambda =1$, corresponding to the normalized eigenvector $v_0$. Since on the other hand $\rho (M(0))\le \left\| M(0)\right\| \le 1$, the Perron–Frobenius theorem implies that $\rho (M(0))=1$.

Moreover, the function ${\mathbb {R}} \rightarrow {\mathbb {R}}:z \mapsto \rho (M(z))$ is strictly decreasing. Indeed, recall that $\rho (M(z)) = \lim _{k \rightarrow \infty } \Vert M(z)^k\Vert ^{1/k}$, where $\Vert \cdot \Vert$ is the matrix norm induced by the standard euclidean norm. All the entries of the matrix *M*(*z*) are strictly decreasing as a function of $z\in \mathbb {R}$, which implies the claim.

Thus, we infer $$\rho (M(z) ) <1 $$ for $$z >0 $$ while $$\rho (M(z)) >1 $$ for $$z < 0$$. Furthermore, by the Perron–Frobenius theorem all eigenvectors with positive entries are associated to the eigenvalue given by the spectral radius $$\rho (M(z))$$. Consequently, $$(0, v_0)$$ is the unique real eigen-solution of (55). $$\square $$

### Theorem 5.2

(Long-time behaviour) Let the assumptions of Lemma 5.1 hold. Let $$v_0 $$ be as in Lemma 5.1. Denote with $$B \in L^1(\mathbb {R}_+;{\mathbb {R}}_+^{|X|})$$ the solution of Eq. (3). Then there exist three constants $$c_0\ge 0 $$, $$ \varepsilon ,\, C >0$$ such that

$$\begin{aligned} \Vert B(t) - c_0 v_0 \Vert \le C e^{- \varepsilon t } \end{aligned}$$

for all $t >0$. Here, $c_0$ depends only on the vector $\int _0^\infty B^0(s)\, ds$.

Before starting the proof of Theorem 5.2 we state Wielandt’s theorem (see Carl (2000), Chapter 8.3). This theorem will be used in the proof of Theorem 5.2.

### Theorem 5.3

(Wielandt’s theorem) Let $A, B \in {\mathbb {C}}^{n \times n}$. If $B \le A$ where *A* us irreducible, then $\rho (B) \le \rho (B)$. If equality holds (i.e. if $\mu = \rho (A) e^{i \phi } \in \sigma (B)$ for some $\phi \in {\mathbb {R}}$, then

$$\begin{aligned} B=e^{i \phi }DAD^{-1} \text { for some } D=\operatorname {diag}\{ e^{i \theta _i }\}_{i=1}^n. \end{aligned}$$

### Proof of Theorem 5.2

By the Laplace inversion formula, see Diekmann et al. (2012), applied to (54) we have that

56 $$\begin{aligned} B(t)= \frac{1}{2 \pi i } \lim _{ T \rightarrow \infty } \int _{\gamma -iT}^{\gamma +iT} e^{z t } (I-M(z))^{-1} \hat{B}^0 (z) dz \end{aligned}$$

for $$\gamma > \sup \{ Re z: \det (I-M(z) )=0\} $$.

We now compute the integral on the right-hand side of Eq. (56). To this end, we first prove that there exists an $\varepsilon >0$ such that, if $z \in {\mathbb {C}}$ satisfies $\det (I-M(z) )=0$, then $\Re z \le - \varepsilon$ or $z=0$. Indeed assume that there exists a ${\overline{z}} \in {\mathbb {R}}\setminus \{0\}$ such that $\det (I-M( i {\overline{z}} ) )=0$. In particular, $M( i {\overline{z}} )$ has one as eigenvalue. Note that the absolute value of any element of the matrix $M(i{\overline{z}} )$ is smaller or equal than the corresponding element of *M*(0) . Consequently, by Wielandt’s theorem there is a diagonal unitary matrix *D* such that $M(i {\overline{z}})=DM(0) D^{-1}$. More precisely, the diagonal elements of *D* have the form $D_{\alpha \alpha }=e^{i \mu _\alpha }$ for some $\mu _\alpha \in \mathbb {R}$. We infer $M(i {\overline{z}} )_{\alpha \beta }= e^{ i (\mu _\alpha - \mu _\beta ) } M(0)_{\alpha \beta }$. As a consequence we have

$$\begin{aligned} \int _0^\infty \left( 1- e^{ i ( \mu _\alpha - \mu _\beta - {\overline{z}} t ) } \right) \Phi _{\alpha \beta }(t) dt =0. \end{aligned}$$

Since ${\overline{z}}\ne 0$ we obtain $\Phi _{\alpha \beta } (t)=0$ a.e., which contradicts the assumption that the matrix *M*(*z*) is irreducible. We deduce that $\det (I- M(z))=0$ implies $\Re z <0$ or $z=0$.

We now prove that there exists an $\varepsilon$ such that if $\det (I - M(z))=0$ then $\Re z \le - \varepsilon$ or $z=0$. Thanks to the Riemann Lebesgue Lemma for every $x <0$ there exists a $\eta _0 >0$ such that the matrix $(I-M(s+ i \eta ))$ is invertible for every $s \in [ x, 0]$ and $|\eta |> \eta _0$. Moreover, since the function $z \mapsto (I- M(z))^{-1}$ is meromorphic, we deduce that the number of poles contained in the compact set $\{ \lambda \in {\mathbb {C}}: |\Im \lambda | \le \eta _0 \text { and } \Re \lambda \in [ x, 0 ]\}$ is finite. Hence, we can choose $\varepsilon >0$ small enough so that $z \mapsto (I - M(z))^{-1}$ has only one pole $z=0$ in the set $\{ \lambda \in {\mathbb {C}}: \Re \lambda > -\varepsilon \}$.

Therefore we can consider any $\gamma >0$ in (56). We deduce that

$$\begin{aligned} \int _{\gamma -iT}^{\gamma +iT} e^{z t } (I-M(z))^{-1} \hat{B}^0 (z) dz&= \oint _{\Gamma } e^{z t } (I-M(z))^{-1} \hat{B}^0 (z) dz \\&\quad - \sum _{i=1}^3 \int _{\Gamma _i } e^{z t } (I-M(z))^{-1} \hat{B}^0 (z) dz \end{aligned}$$

where $\Gamma := \cup _{i =1}^4\Gamma _i$ and where $\Gamma _4$ is the segment in the complex plane connecting $\gamma +i T$ to $\gamma -i T$, $\Gamma _2$ is the segment connecting $\gamma +i T$ to $-\varepsilon + i T$, $\Gamma _3$ is the segment connecting $-\varepsilon + i T$ to $-\varepsilon - i T$, finally $\Gamma _1$ connects $- \varepsilon -i T$ to $\gamma -i T$.

By the residue theorem we have

$$\begin{aligned} \frac{1}{2 \pi i } \oint _{\Gamma } e^{z t } (I-M(z))^{-1} \hat{B}^0 (z) dz = Res_{z=0} (I-M(z))^{-1} \hat{B}^0 (z). \end{aligned}$$

We now compute $Res_{z=0} (I-M(z))^{-1} \hat{B}^0 (z)$. To this end, we notice that for small *z* we have the following Laurent series representation of $(I - M (z))^{-1}$

$$\begin{aligned} (I - M (z))^{-1} = \sum _{n=-p}^\infty z^n {\mathcal {R}}_n, \end{aligned}$$

where $${\mathcal {R}}_n\in \mathbb {R}^{|X|\times |X|}$$, $$p\ge 1$$. Since $$(I - M (z))^{-1} (I - M (z))=(I - M (z)) (I - M (z))^{-1} = I $$ we deduce that $$ (I - M(z)) {\mathcal {R}}_{-1} ={\textbf {0}}$$ which implies that $${\mathcal {R}}_{-1} v = \alpha v_0 $$ for every vector *v* and for some $$\alpha \in {\mathbb {C}}$$ that depends on *v*. Thus $$Res_{z=0}(I-M(z))^{-1} \hat{B}^0 (z) = {\mathcal {R}}_{-1} \hat{B}^0 (0)= c_0 v_0$$ where the constant $$c_0\ge 0$$ depends on $$\hat{B}^0(0)= \int _0^\infty B^0(s)\, ds$$. In addition, by the Riemann Lebesgue Lemma, we have that

$$\begin{aligned} \lim _{T \rightarrow \infty } \frac{1}{2 \pi i } \left\| \int _{\Gamma _i} e^{z t } (I-M(z))^{-1} \hat{B}^0 (z) dz \right\| =0 \end{aligned}$$

for $$i=1,2$$. Moreover

$$\begin{aligned}&\lim _{T \rightarrow \infty } \frac{1}{2 \pi i } \left\| \int _{\Gamma _3 } e^{z t } (I-M(z))^{-1} \hat{B}^0 (z) dz \right\| \\&\quad = \lim _{T \rightarrow \infty } \frac{1}{2 \pi i } \left\| \int _{- \varepsilon - i T }^{- \varepsilon + i T } e^{z t } (I-M(z))^{-1} \hat{B}^0 (z) dz \right\| \le C e^{-\varepsilon t }. \end{aligned}$$

The desired conclusion follows.

$$\square $$

### Lemma 5.4

Make the assumptions of Theorem 5.2. Let $\textbf{K} \in {\mathbb {R}}_+^{|X| \times |X|}$ be the diagonal matrix s.t. $\textbf{K}_{\alpha \alpha } = k_\alpha$. Denote with $D \in {\mathbb {R}}_+^{|X|}$ the function given by (4). Assume that there exist $z_0, C_1, C_2 >0$ such that for every $t>0$

$$\begin{aligned} k_\alpha \le C_1 e^{- z_0 t } \text { and } D^0_\alpha \le C_2 e^{- z_0 t } \end{aligned}$$

for every $\alpha \in X$. Then there exist two constants $C, \rho >0$ such that

$$\begin{aligned} \left\| D(t) - c_0 v_0 \right\| \le Ce^{-\rho t } \end{aligned}$$

for every $$t >0$$, where the constant $$c_0 $$ is the same as in Theorem 5.2.

### Proof

First of all, note that $\int _0^\infty k_\alpha (t ) dt =1$ for every $\alpha \in X$ since $\{\Phi \}$ is irreducible. Then, by definition of *D*(*t*) and the bounds on $D^0$, $k_\alpha$ we have

$$\begin{aligned} \left\| D (t) - c_0 v_0 \right\|&= \left\| D^0 (t) + \int _0^t {\textbf {K}} (s) B(t-s) ds - c_0 \int _0^t {\textbf {K}}(s) v_0ds - c_0 \int _t^\infty {\textbf {K}}(s) v_0ds \right\| \\&\le C e^{- z_0 t }+ \left\| \int _0^t {\textbf {K}}(s) \left[ B(t-s) - c_0 v_0\right] ds \right\| \le C e^{- z_0 t } \\&\quad + \int _0^t \left\| {\textbf {K}}(s) \right\| \left\| B(t-s) - c_0 v_0\right\| ds \le C e^{- z_0 t }+ C e^{- \varepsilon t }\int _0^t \left\| {\textbf {K}}(s) \right\| e^{ \varepsilon s } ds \\&\le C e^{- z_0 t }+ C e^{- \varepsilon t }\int _0^t e^{ ( \varepsilon - z_0) s } ds \le C e^{- \rho t } \end{aligned}$$

for some $\rho >0$. Notice that for the above computation we have used the long-time behaviour of *B* computed in Theorem 5.2 and the bounds on $k_\alpha$ and $D^0_\alpha$. $\square$

### Theorem 5.5

Make the assumptions of Theorem 5.2. Let $\textbf{K} \in {\mathbb {R}}_+^{|X|}$ be the diagonal matrix s.t. $\textbf{K}_{\alpha \alpha } = k_\alpha$. Assume in addition that $N^0= \int _0^\infty D^0(s) ds$. Then the solution $N (t) \in {\mathbb {R}}_+^{|X|}$ of (2) is such that there exists two positive constants *r* and *C* such that for all $t>0$

57 $$\begin{aligned} \left\| N(t) - c_0 v_0 \int _0^\infty s \textbf{K}(s) ds \right\| \le C e^{-rt }, \end{aligned}$$

where $c_0$ is the same constant as in Theorem 5.2.

### Proof

By definition of $N=(N_\alpha )_{\alpha \in X}$ we have that

$$\begin{aligned} N(t)=N^0 + \int _0^t \left[ B(s) - D(s) \right] ds. \end{aligned}$$

Then

$$\begin{aligned}&\left\| N(t) - c_0 v_0 \int _0^\infty v \textbf{K} (v) dv \right\| \\&\quad = \left\| N^0 + \int _0^t \left[ B(s) - D(s) \right] ds - c_0 v_0 \int _0^\infty v \textbf{K} (v) dv \right\| \\&\quad = \left\| N^0 + \int _0^t \left[ B(s) - D^0(s) - \int _0^s \textbf{K}(s-v ) B(v) dv \right] ds - c_0 v_0 \int _0^\infty v \textbf{K} (v) dv \right\| \\&\quad \le \left\| N^0 - \int _0^t D^0(s) ds \right\| \\&\qquad + \left\| \int _0^t \left[ B(s) - \int _0^s \textbf{K}(s-v ) B(v) dv \right] ds -c_0 v_0 \int _0^\infty v \textbf{K} (v) dv \right\| . \end{aligned}$$

We know that, for some positive constants $C_1, \, C_2, \, C_3$ and $z_0,\, \varepsilon >0$

$$\begin{aligned} \left\| N^0 - \int _0^t D^0(s) ds \right\|&\le C_1 e^{-z_0 t }, \ \left\| B(t) - c_0 v_0 \right\| \le C_2 e^{-\varepsilon t }, \ \left\| v_0 \int _t^\infty \int _s^\infty {\textbf {K}} (v) dv ds \right\| \\&\le C_3 e^{-z_0 t}. \end{aligned}$$

Then, we conclude

$$\begin{aligned}&\left\| N^0 - \int _0^t D^0(s) ds \right\| + \left\| \int _0^t \left[ B(s) - \int _0^s \textbf{K}(s-v ) B(v) dv \right] ds - c_0v_0 \int _0^\infty v \textbf{K}(v) dv \right\| \\&\quad \le (C_1+C_3)e^{-z_0 t } + \left\| \int _0^t B(s)\int _{t-s}^\infty \textbf{K}(v ) dv ds -c_0 v_0 \int _0^t \int _{t-s}^\infty \textbf{K} (v) dv ds \right\| \\&\quad \le Ce^{-z_0 t } + \left\| \int _0^{t} \left[ B(s) -c_0 v_0 \right] \int _{t-s}^\infty \textbf{K}(v ) dv ds \right\| \le C e^{-z_0 t } \\&\qquad + \int _0^{t} \left\| B(s) -c_0 v_0 \right\| \left\| \int _{t-s}^\infty \textbf{K}(v ) dv \right\| ds \le C e^{-z_0 t } + C \int _0^{t} e^{-\varepsilon s} e^{(s-t) z_0 } ds \le C e^{-rt} \end{aligned}$$

for some $$r,\, C>0$$. $$\square $$

## Response functions and structured population equations

In this section, we study the relation between the formalism of response functions introduced in Sect. 2 and the formalism of structured population models, which is often used in biology, see for instance (Perthame 2006). The theory of structured population models is a very well devolped field. Therefore relating structured population equations to RFEs can help the understanding of RFEs. Moreover as explained in the introduction the RFEs are, in general, non-Markovian equations, while SPEs are Markovian. In Sect. 6.1 we study the relation between (2)–(4) and (9), while in Sect. 6.2 we consider the equivalence between (16)–(18) and generalizations of (9). We notice that in order to obtain this equivalence it is crucial to study the properties of the forcing function $\{ B_\alpha ^0 \}$ which describes the transient response of the system, (cf. 67). Finally, in Sect. 6.3 we describe the conditions on the initial data $\{ n_\alpha ^0\}$ that allow to rewrite the ODE model (15) as SPEs.

### Classical RFEs

Here, we reformulate (2)–(4) as a structured population model and we study the equivalence of the two formulations. To this end, we introduce the concept of age of an individual in some compartment $\alpha$. In this section, we assume that every element enters the compartment $\alpha$ with the same age $\xi =0$.

Let $f_\alpha (t, \xi )$ be the number of elements in the compartment $\alpha$ with age $\xi$ at time *t*. Since here we assume that all the elements enter the compartment $\alpha$ with age $\xi =0$, the age of an element in the compartment $\alpha$ at a certain time $t \ge 0$ is just $\xi =t-{\overline{t}}$, where ${\overline{t}}$ is the time at which it entered in $\alpha$.

We then have the following evolution equation for $f_\alpha$

58 $$\begin{aligned}&\partial _t f_\alpha (t,\xi ) + \partial _\xi f_\alpha (t,\xi ) = - \Lambda _\alpha (\xi ) f_\alpha (t,\xi ) , \end{aligned}$$

59 $$\begin{aligned}&f_\alpha (t,0) = \sum _{\beta \in X \setminus \{\alpha \} } \int _{\mathbb {R}_+} \lambda _{ \beta \alpha } (\eta ) f_\beta (t,\eta ) d\eta \end{aligned}$$

60 $$\begin{aligned}&f_\alpha (0,\xi )=m_\alpha (-\xi ) e^{- \int _0^{\xi } \Lambda _\alpha (s) ds } \end{aligned}$$

where $\Lambda _\alpha (\xi ) = \sum _{\beta \in X \setminus \{\alpha \} }\lambda _{\alpha \beta }(\xi )$. Indeed, $f_\alpha (t, \xi )$ changes in time due to the aging of the elements, described by the transport term in (58), due to jumps from the compartment $\alpha$ to any other compartment $\beta \in X$, described by the loss term in (58), and, finally, due to the fact that elements enter the compartment $\alpha$ with age zero, from any other compartment $\beta \in X$. Accordingly, $\lambda _{\alpha \beta } (\eta )$ in the above equation is the rate at which an individual, which has been in the compartment $\alpha$ for a time interval of length $\eta$, jumps from compartment $\alpha$ to compartment $\beta$. Finally, $m_\alpha (-\xi )$ in (60) is the number of elements with state in the compartment $\alpha$ at time $-\xi$. We then obtain $f_\alpha (0, \xi )$, multiplying $m_\alpha (\xi )$ by the probability that these elements stay in the compartment $\alpha$ up to time 0, namely multiplying by $e^{-\int _0^\xi \Lambda _\alpha (s) ds }$.

#### Definition 6.1

Let $\lambda _{\alpha \beta } \in C_b({\mathbb {R}}_+ )$ be non-negative for every $\alpha , \beta \in X$. Assume that $f_\alpha ^0 \in {\mathcal {M}}_{+, b} ({\mathbb {R}}_+)$ for every $\alpha \in X$. A family of functions $\{ f_\alpha \}$ with $f_\alpha \in C([0, T]; {\mathcal {M}}_{+,b}({\mathbb {R}}_+) )$ is a solution of Eq. (58), with initial condition $f_\alpha ^0(\cdot )$, if for every $\varphi \in C^1_c({\mathbb {R}}_+)$ the map $t \mapsto \int _{{\mathbb {R}}_+} \varphi (\xi ) f_\alpha (t, d \xi )$ is differentiable and

61 $$\begin{aligned} \frac{d}{dt} \int _{{\mathbb {R}}_+} \varphi (\xi ) f_\alpha (t, d \xi )&= \int _{{\mathbb {R}}_+} \left[ \varphi '(\xi ) - \Lambda _\alpha (\xi ) \varphi (\xi ) ) \right] f_\alpha (t , d \xi ) \end{aligned}$$

62 $$\begin{aligned}&\quad + \varphi (0) \sum _{\beta \in X \setminus \{ \alpha \} } \int _{{\mathbb {R}}_+} \lambda _{\beta \alpha } (\eta ) f_\beta (t, d\eta ) \end{aligned}$$

for every $\alpha \in X$. Furthermore $f_\alpha (0, \cdot )=f_{\alpha }^0(\cdot )$.

We recall that in the definition of $C([0, T], {\mathcal {M}}_{+, b} ({\mathbb {R}}_+))$ we endow the space ${\mathcal {M}}_{+, b} ({\mathbb {R}}_+)$ with the Wasserstein distance, see Sect. 1.1.

#### Lemma 6.1

Let $\lambda _{\alpha \beta } \in C_b({\mathbb {R}}_+ )$ to be non-negative for every $\alpha , \beta \in X$. Let $m_\alpha \in {\mathcal {M}}_{+, b} ({\mathbb {R}}_+)$ for $\alpha \in X$. Assume that $f_\alpha ^0$ is given by (60). Then there exists a unique solution $\{ f_\alpha \}$ with $f_\alpha \in C([0, T], {\mathcal {M}}_{+, b}({\mathbb {R}}_+) )$ for all $\alpha \in X$ to Eq. (58) in the sense of Definition 6.1. The solution satisfies

63 $$\begin{aligned} \int _{{\mathbb {R}}_+} e^{ \int _0^{\xi } \Lambda _\alpha (v) d v } f_\alpha (t, d \xi ) < \infty , \end{aligned}$$

for every $\alpha \in X$ and every $t \ge 0$, where $\Lambda _\alpha (t):= \sum _{\beta \in X \setminus \{\alpha \} } \lambda _{\alpha \beta } (t)$.

#### Proof

We can rewrite Eq. (61) in fixed point form as follows. We use the notation $Y:=C([0, T], {\mathcal {M}}_{+,b}({\mathbb {R}}_{+} ) )^{|X| }$ where *Y* is endowed with the metric induced by the distance

$$\begin{aligned} d_T(f,g)= \sum _{\alpha \in X} \sup _{t \in [0, T] } W_1( f_\alpha (t, \cdot ) g_\alpha (t, \cdot ) ). \end{aligned}$$

Given a $f=( f_\alpha ) \in Y$ we define the operator ${\mathcal {T}} [f](t): C_c({\mathbb {R}}_+) \rightarrow {\mathbb {R}}_+^{|X|}$ as

64 $$\begin{aligned} \langle {\mathcal {T}} [f] (t) , \varphi \rangle _\alpha&:= \int _0^t \varphi (t- \xi ) e^{- \int _0^{t- \xi } \Lambda _\alpha (v) dv } \sum _{\beta \in X \setminus \{ \alpha \} } \int _{{\mathbb {R}}_+} \lambda _{\beta \alpha } (\eta ) f_\beta (\xi , d \eta ) d \xi \nonumber \\&\quad + \int _{{\mathbb {R}}_+} f_\alpha ^0(d\xi ) \varphi (\xi +t) e^{- \int _\xi ^{t+\xi } \Lambda _\alpha (v) dv } \end{aligned}$$

where we are using the notation $\langle \cdot , \cdot \rangle _\alpha$ to indicate the $\alpha$-th component of ${\mathcal {T}}[f] (t)$ applied to $\varphi$.

We define $\Vert f \Vert _Y:=\sum _{\alpha \in X} \sup _{t \in [0, T]} \Vert f_\alpha (t, \cdot ) \Vert _{TV}$ and the set ${\mathcal {X}}_T \subset Y$ as

$$\begin{aligned} {\mathcal {X}}_T:= \left\{ f \in Y: (f(0))_\alpha =f_\alpha ^0 \ \text { for every } \alpha \in X, \ ||f ||_Y \le 1 + \sum _{\alpha \in X} \Vert f^0_\alpha \Vert _{TV} \right\} . \end{aligned}$$

For every $f \in {\mathcal {X}}_T$ each component of the operator ${\mathcal {T}}[f](t)$ is a linear, positive and continuous operator from $C_c({\mathbb {R}}_+)$ to ${\mathbb {R}}$ and, hence, can be identified with an element of ${\mathcal {M}}_{+, b} ({\mathbb {R}}_+).$

Using the bound for the parameters $\lambda _{\alpha \beta }$, we deduce that, for every $f \in {\mathcal {X}}_T$, we have that ${\mathcal {T}} [f] \in {\mathcal {X}}_T$ for sufficiently small values of *T*. Similarly, for sufficiently small values of *T*, the operator ${\mathcal {T}}$ is a contraction and hence Banach fixed point Theorem implies that there exists a unique fixed point $f \in {\mathcal {X}}_T$.

Since the fixed point *f* of ${\mathcal {T}}$ satisfies ${\mathcal {T}} [f](t) = f (t)$ the map $t \mapsto \langle f(t, \cdot ), \varphi \rangle _\alpha =\langle {\mathcal {T}}[f] (t), \varphi \rangle _\alpha$ is differentiable if $\varphi \in C^1_c({\mathbb {R}}_+)$. Hence, the fixed point of ${\mathcal {T}}$ is a solution to (61) for *T* small enough. Due to the linearity of the problem $\Vert f \Vert _Y$ stays finite for every $t>0.$ We can then iterate the argument and prove the existence and uniqueness of a solution to (61) for an arbitrary $T>0$.

To prove (63) we consider the test function $\varphi _{\varepsilon , R}(\xi )= \chi _{R, \varepsilon } (\xi )e^{\int _0^\xi \Lambda _\alpha (v) dv}$ in Eq. (64). Here $\chi _{R, \varepsilon }$ is a smooth decreasing function such that $\chi _{R, \varepsilon } (\xi ) = 0$ for $\xi > R+ \varepsilon$ and $\chi _{R, \varepsilon } (\xi ) = 1$ for $\xi \le R$. We deduce that

$$\begin{aligned}&\int _{{\mathbb {R}}_+} \chi _{R,\varepsilon }(\xi ) e^{ \int _0^\xi \Lambda _\alpha (v) dv } f_\alpha (t, d\xi ) \le \int _0^t \chi _{R,\varepsilon }(t- \xi ) \sum _{\beta \in X \setminus \{ \alpha \} } \int _{{\mathbb {R}}_+} \lambda _{\beta \alpha } (\eta ) f_\beta (\xi , d \eta ) d \xi \\&\quad + c_1 f^0_\alpha ({\mathbb {R}}_+) \le C \sup _{t \in [0, T] } \sum _{ \beta \in X \setminus \{ \alpha \}} f_\beta (t, {\mathbb {R}}_+) + c_1 f^0_\alpha ({\mathbb {R}}_+) \end{aligned}$$

where the positive constants $c_1, C$ do not depend on $\varepsilon$ and *R*. Sending $\varepsilon$ to zero and *R* to infinity concludes the proof. $\square$

Since we are assuming that each of the compartments $\alpha \in X$ has at most one entrance point, we can consider the simplified system for $\{N_\alpha , B_\alpha , D_\alpha \}$, cf. (2), (3), (4). In the following two theorems, we study the equivalence between (58)–(60) and (2)–(4).

#### Theorem 6.2

(RF and SP) Under the assumptions of Lemma 6.1 consider the solution $\{ f_\alpha \}$ with $f_\alpha \in C([0, T], {\mathcal {M}}_{+, b}({\mathbb {R}}_{+}))$ to (58)–(60). Then, the family of functions $\{ N_\alpha \}$, defined by

65 $$\begin{aligned}&N_\alpha (t)= \int _{{\mathbb {R}}_+}f_\alpha (t,d \xi ), \quad t\ge 0 \quad \forall \alpha \in X, \end{aligned}$$

satisfies (16) where $\{B_\alpha , D_\alpha \}$ satisfy (3), (4) with response functions $\{\Phi _{\alpha \beta } \}$ and $\{k_\alpha \}$ given by

66 $$\begin{aligned} \Phi _{\alpha \beta } (t)= \lambda _{\alpha \beta }(t)e^{- \int _0^t \Lambda _\alpha (s) ds } , \quad k_\alpha (t)= \Lambda _\alpha (t)e^{- \int _0^t \Lambda _\alpha (s) ds } \end{aligned}$$

and with

67 $$\begin{aligned} B^0_\alpha (t) = \sum _{\beta \in X \setminus \{ \alpha \} } \int _{{\mathbb {R}}_- } \Phi _{ \beta \alpha }(t-\xi ) m_\beta ( d\xi ), \quad D^0_\alpha (t) = \int _{{\mathbb {R}}_-} k_\alpha (t-\xi ) m_\alpha ( d\xi ). \end{aligned}$$

#### Proof

From Eq. (61) we deduce that

$$\begin{aligned} \frac{d}{dt} N_\alpha (t)= \frac{d}{dt} \int _{{\mathbb {R}}_+} f_\alpha (t, d\xi ) = - \int _{{\mathbb {R}}_+} \Lambda _\alpha (\xi ) f_\alpha (t, d\xi ) + \sum _{\beta \in X \setminus \{\alpha \} } \int _{{\mathbb {R}}_+ } \lambda _{\beta \alpha } (\xi ) f_\beta ( t, d \xi ). \end{aligned}$$

Notice that we can use the test function $\varphi =1$ due to (63).

The fixed point formulation of Eq. (61), namely (64), implies that

68 $$\begin{aligned} \int _{{\mathbb {R}}_+} \lambda _{\beta \alpha } (\xi ) f_\beta (t, d\xi )&= \int _0^t \lambda _{\beta \alpha }(t-\xi ) e^{- \int _0^{t-\xi } \Lambda _\beta (v) dv } \sum _{\gamma \in X \setminus \{ \beta \} } \int _{{\mathbb {R}}_+} \lambda _{\gamma \beta } (\eta ) f_\gamma (\xi , d \eta ) d \xi \nonumber \\&\quad + \int _{{\mathbb {R}}_-}\lambda _{\beta \alpha } (t-\xi ) e^{- \int _{0}^{t-\xi } \Lambda _\beta (v) dv} m_\beta (d \xi ) . \end{aligned}$$

Summing in the above equation over $\beta \in X \setminus \{ \alpha \}$ we deduce that

$$\begin{aligned}\sum _{\beta \in X \setminus \{\alpha \} } \int _{{\mathbb {R}}_+ } \lambda _{\beta \alpha } (\xi ) f_\beta ( t, d \xi )=:S_\alpha (t) \end{aligned}$$

satisfies (3) with kernel $\Phi _{\alpha \beta }$ as in (66) and with the forcing function $B^0_\alpha$ given by (67). Similarly, taking the sum over $\alpha \in X \setminus \{ \beta \}$ we deduce that

$$\begin{aligned} \int _{{\mathbb {R}}_+} \Lambda _\alpha (\xi ) f_\alpha (t, d\xi ) = \int _0^t k_\alpha ( t-\xi ) \sum _{\gamma \in X \setminus \{\alpha \} } \int _{{\mathbb {R}}_+} \lambda _{\gamma \alpha } (\eta ) f_\gamma (\xi , d \eta ) d\xi + D^0_\alpha (t), \end{aligned}$$

where $k_\alpha$ is as in (66). Hence,

$$\begin{aligned} D_\beta ^0 (t) = \int _{{\mathbb {R}}_+} \Lambda _\beta (\xi ) f_\beta (t, d\xi ) \end{aligned}$$

satisfies (4). This concludes the proof. $\square$

#### Theorem 6.3

(RF and SP) Let $\{N_\alpha , B_\alpha , D_\alpha \}$ satisfy (2)–(4) with response functions $\Phi _{\alpha \beta } \in C({\mathbb {R}}_+; {\mathbb {R}}_+)$ and $k_\alpha \in C({\mathbb {R}}_+; {\mathbb {R}}_+)$ and assume that $\{B_\alpha ^0, D_\alpha ^0 \}$ satisfy (67) where $\{ m_\alpha \}$ is a family of measures $m_\alpha \in {\mathcal {M}}_{+, b}({\mathbb {R}}_+)$. Assume in addition that for every $\alpha , \beta \in X$ it holds that the function

69 $$\begin{aligned} \lambda _{\alpha \beta }(t):= \frac{\Phi _{\alpha \beta }(t) }{1- \sum _{\gamma \in X \setminus \{ \alpha \} } \int _0^t \Phi _{\alpha \gamma }(s) ds } \quad t \ge 0 \end{aligned}$$

is continuous and bounded for every $\alpha , \beta$. Then the family of functions $\{ f_\alpha \}$ solving (58)–(60) satisfy (65).

Before proving the theorem we remark that the continuity of $\lambda _{\alpha \beta }$ as in (69) holds if we assume that for every $\alpha ,\beta \in X$ the support of the function $\Phi _{\alpha \beta }$ is unbounded. Notice that this assumption is true for all the examples in Sect. 7.

#### Proof

Since $\{f_\alpha \}$ solves (58)–(60) by Theorem 6.2 the functions $\bar{N}_\alpha (t):= \int _{\mathbb {R}_+}f_\alpha (t,d\xi )$ solve (2)–(4) with the same initial conditions as $\{N_\alpha ,B_\alpha ,D_\alpha \}$ and some response functions $\{\bar{\Phi }_{\alpha \beta }\}$ given by (66). Since $\Lambda _\alpha$ is given by

$$\begin{aligned} \Lambda _\alpha (t)= \frac{ \sum _{\beta \in X \setminus \{\alpha \} }\Phi _{\alpha \beta }(t) }{1- \sum _{\gamma \in X \setminus \{\alpha \} } \int _0^t \Phi _{\alpha \gamma }(s) ds }, \end{aligned}$$

then we have that

$$\begin{aligned} \int _0^t \Lambda _\alpha (v) dv&= \int _0^t \frac{ \sum _{\beta \in X \setminus \{\alpha \} }\Phi _{\alpha \beta }(v) }{1- \sum _{\gamma \in X \setminus \{\alpha \} } \int _0^v \Phi _{\alpha \gamma }(s) ds } dv\\&= - \ln \left( 1- \int _0^t \sum _{\beta \in X \setminus \{\alpha \} }\Phi _{\alpha \beta }(s) ds \right) . \end{aligned}$$

Hence,

70 $$\begin{aligned} e^{- \int _0^t \Lambda _\alpha (v) dv } = 1- \int _0^t \sum _{\beta \in X \setminus \{\alpha \} }\Phi _{\alpha \beta }(s) ds. \end{aligned}$$

By (66) we deduce that ${\bar{\Phi }}_{\alpha \beta } = \Phi _{\alpha \beta }$. By uniqueness of the solution, we obtain $N_\alpha =\bar{N}_\alpha$. This concludes the proof. $\square$

### Generalized RFEs

Here, under suitable conditions on the forcing functions, we reformulate (16)–(18) as a structured population model. As in Sect. 6.1, we assume that the elements have age $\xi =0$ when they enter in the compartment. We assume that $f_{\alpha j} (t, \xi )$ is the density of individuals that entered the compartment $\alpha$ with state $j \in \alpha$, and that have age $\xi$ at time *t*.

The evolution in time of $f_{\alpha j }$ is described by the following SPEs

71 $$\begin{aligned}&\partial _t f_{\alpha j } (t,\xi ) + \partial _\xi f_{\alpha j } (t, \xi ) = - M_{\alpha j}(\xi ) f_{\alpha j } (t, \xi ) \end{aligned}$$

72 $$\begin{aligned}&f_{\alpha j } ( t, 0)= \sum _{\beta \in X \setminus \{\alpha \} } \sum _{ k \in \beta } \int _{\mathbb {R}_+} f_{\beta k } (t , \eta ) \mu _{kj}(\eta ) d \eta \end{aligned}$$

73 $$\begin{aligned}&f_{\alpha j } ( 0, \xi ) = m_{\alpha j } (- \xi ) e^{- \int _0^\xi M_{\alpha j } (v) dv } \end{aligned}$$

where $$M_{\alpha j} (\xi )= \sum _{\beta \in X \setminus \{\alpha \} } \sum _{\ell \in \beta } \mu _{j \ell } (\xi )$$.

The transport term in (71) is due to the aging of the elements in the compartments. Moreover, elements with "state-at-entrance" $j \in \alpha$ and age $\xi$ jump to another compartment with rate $M_{\alpha j } (\xi )$. The birth term in (72) is due to elements that entered $\beta$ with any state and at any time in the past, that at time *t* jump to state $j \in \alpha$. Finally $m_\alpha (-\xi )$ in (71) is a vector whose *j*-th element is the density of elements with state $j \in \alpha$ at time $-\xi$.

Eq. (71)–(73) is a special case of (58)–(60). Indeed, consider in (58)–(60) a set of compartments $X_1$ such that every compartment in $X_1$ is of the form $\{ j \}$ with $j \in \Omega$. Moreover, we assume that the compartments $\{ j\}$ with $j \in \alpha$ with $\alpha \in X$, are not connected, which means that $\lambda _{ij} =0$ when $i, j \in \alpha$. Then (58)–(60) for the set of compartments $X_1$ reduce to (71)–(73) for the set of compartments *X*.

We therefore define a solution of (71)–(73) in analogy with Definition 6.1.

#### Definition 6.2

Let $\mu _{ij } \in C_b({\mathbb {R}}_+ )$ be non-negative for every $i \in \alpha$, $j \in \beta$ with $\alpha , \beta \in X$. Assume that $f_{\alpha j}^0 \in {\mathcal {M}}_{+, b} ({\mathbb {R}}_+)$ for every $j \in \alpha$ and every $\alpha \in X$. A family of functions $\{ f_{\alpha j} \}$ with $f_{\alpha j} \in C([0, T]; {\mathcal {M}}_{+,b}({\mathbb {R}}_+) )$ is a solution of Eq. (71), with initial condition $f_{\alpha j } ^0(\cdot )$, if for every $\varphi \in C^1_c({\mathbb {R}}_+)$ the map $t \mapsto \int _{{\mathbb {R}}_+} \varphi (\xi ) f_{\alpha j} (t, d \xi )$ is differentiable and

74 $$\begin{aligned} \frac{d}{dt} \int _{{\mathbb {R}}_+} \varphi (\xi ) f_{\alpha j } (t, d \xi ) =&\int _{{\mathbb {R}}_+} \left[ \varphi '(\xi ) - M_{\alpha j } (\xi ) \varphi (\xi ) ) \right] f_{\alpha j } (t , d \xi ) \nonumber \\&+ \varphi (0) \sum _{\beta \in X \setminus \{ \alpha \} } \sum _{k \in \beta } \int _{{\mathbb {R}}_+} \mu _{k j } (\eta ) f_{\beta k } (t, d\eta ) \end{aligned}$$

for every $j \in \alpha$ with $\alpha \in X$. Furthermore $f_{\alpha j } (0, \cdot )=f_{\alpha j }^0(\cdot )$.

Moreover, as a consequence of Lemma 6.1 we have the existence of a unique solution for (71)–(73).

#### Lemma 6.4

Let $\mu _{ij } \in C_b({\mathbb {R}}_+ )$ to be non-negative for every *i*, *j*. Let $m_{\alpha j } \in {\mathcal {M}}_{+, b} ({\mathbb {R}}_+)$ for $\alpha \in X$. Assume that $f_{\alpha j}^0$ is given by (60). Then there exists a unique solution $\{ f_{\alpha j } \}$ with $f_{ \alpha j } \in C([0, T], {\mathcal {M}}_{+, b}({\mathbb {R}}_+) )$ for all $j \in \alpha$ and $\alpha \in X$ to Eq. (71) in the sense of Definition 6.2. The solution satisfies, for every $j \in \alpha$ and $\alpha \in X$ and every $t \ge 0$

75 $$\begin{aligned} \int _{{\mathbb {R}}_+} e^{ \int _0^{\xi } M_{ \alpha j } (v) d v } f_{\alpha j } (t, d \xi ) < \infty , \end{aligned}$$

where $$M_{\alpha j } (t):= \sum _{\beta \in X \setminus \{\alpha \} } \sum _{i \in \beta } \mu _{ i j } (t)$$.

In the following two theorems, we study the equivalence between (71)–(73) and (16)–(18).

#### Theorem 6.5

(RF and SP) Under the assumptions of Lemma 6.4 consider the solution $\{ f_{\alpha j } \}$ with $f_{\alpha , j } \in C([0, T], {\mathcal {M}}_{+, b}({\mathbb {R}}_{+}))$ to (71)–(73). Then, the family of functions $\{ N_\alpha \}$, defined by

76 $$\begin{aligned}&N_\alpha (t)= \sum _{j \in \alpha } \int _{{\mathbb {R}}_+}f_{\alpha j } (t,d \xi ), \quad t\ge 0, \quad \forall \alpha \in X, \end{aligned}$$

satisfies (16). The corresponding fluxes $\{ S_\alpha \}$ and $\{J_\alpha \}$ satisfy (17) and (18) with response functions given by

77 $$\begin{aligned} ( G_{\alpha \beta }(t) )_{j k }= \mu _{kj } (t) e^{-\int _0^t M_{\alpha k } (v) dv } \end{aligned}$$

for $$k \in \alpha , j \in \beta $$ and

78 $$\begin{aligned} ( K_\alpha (t) )_{ii } = M_{\alpha i } (t) e^{- \int _0^t M_{\alpha i } (s ) ds }, \quad ( K_\alpha (t) )_{ij } = 0 \end{aligned}$$

for $i, \, j \in \alpha$. The corresponding forcing functions have the form

79 $$\begin{aligned} S^0_\alpha (t)= \sum _{\beta \in X \setminus \{\alpha \}} \int _{{\mathbb {R}}_-} G_{\beta \alpha } (t-s) m_\beta (d s ), \quad J^0_\alpha (t)= \int _{{\mathbb {R}}_-} K_{ \alpha } (t-s) m_\alpha (d s ). \end{aligned}$$

Here, we write $m_\alpha \in \mathbb {R}^{|\alpha |}$, $(m_\alpha )_j = m_{\alpha j }$ for every $j \in \alpha$, $\alpha \in X$.

#### Proof

We have that

$$\begin{aligned} \frac{d}{dt} N_\alpha (t)= - \sum _{j \in \alpha } \int _{{\mathbb {R}}_+} M_{\alpha j} (\xi ) f_{\alpha j }(t, d\xi ) + \sum _{j \in \alpha } \sum _{\beta \in X \setminus \{ \alpha \}} \sum _{ k \in \beta } \int _{{\mathbb {R}}+} \mu _{k j }(\xi ) f_{\beta k } (t, d \xi ). \end{aligned}$$

As in the proof of Theorem 6.2 one can show that the function $t \mapsto S_\alpha (t)$ with $S_\alpha (t)\in {\mathbb {R}}^{|\alpha |}$ given by

$$\begin{aligned} (S_\alpha (t) )_j = \sum _{\beta \in X \setminus \{ \alpha \}} \sum _{ k \in \beta } \int _{{\mathbb {R}}_+} \mu _{kj } (\xi ) f_{\beta k } (t, d \xi ) \end{aligned}$$

satisfies (17) with response function given by (77) and with forcing function given by (79). Similarly, $J_\alpha (t) \in {\mathbb {R}}_+^{|\alpha |}$ given by

$$\begin{aligned} (J_\alpha (t))_j = \int _{{\mathbb {R}}_+} M_{\alpha j } (\xi ) f_{\alpha j } (t, d\xi ) \end{aligned}$$

satisfy (18) with response function (78) and forcing function (79). $\square$

#### Theorem 6.6

(RF and SP) Let $\{N_\alpha , S_\alpha , J_\alpha \}$ satisfy (16)–(18) with response functions $G_{\alpha \beta } \in C({\mathbb {R}}_+; {\mathbb {R}}_+^{|\beta |\times |\alpha |})$ and $K_\alpha \in C({\mathbb {R}}_+; {\mathbb {R}}_+^{|\alpha |\times |\alpha |})$ assume that $\{S_\alpha ^0, J_\alpha ^0 \}$ satisfy (67) where $\{ m_{\alpha j } \}$ is a family of measures $m_{\alpha j} \in {\mathcal {M}}_{+, b}({\mathbb {R}}_+)$. Assume in addition that for every $i \in \alpha$ and $j \in \beta$ with $\alpha , \beta \in X$ the function

80 $$\begin{aligned} \mu _{ij }(t):= \frac{(G_{\alpha \beta }(t))_{ji} }{1- \sum _{\gamma \in X \setminus \{ \alpha \} } \sum _{k \in \gamma } \int _0^t (G_{\alpha \gamma }(s))_{ki } ds } \quad t \ge 0 \end{aligned}$$

is continuous and bounded. Then the family of functions $\{ f_{\alpha i} \}$ solving (71)–(73) satisfies (76).

#### Proof

Since $\{f_{\alpha j} \}$ solves (71)–(73) with coefficients $\mu _{ij}$ given by (80) the functions $\bar{N}_\alpha (t):= \sum _{ j \in \alpha } \int _{\mathbb {R}_+}f_{\alpha j }(t,d\xi )$ satisfy (16)–(18) with the same forcing function as $\{N_\alpha ,S_\alpha ,J_\alpha \}$ and some response functions $\{\bar{G}_{\alpha \beta }\}$ given by (79). Then

$$\begin{aligned} \int _0^t M_{\alpha i } (v) dv&= \int _0^t \frac{ \sum _{\beta \in X \setminus \{\alpha \} } \sum _{j \in \beta } (G _{\alpha \beta }(v))_{ji }}{1- \sum _{\gamma \in X \setminus \{\alpha \} } \sum _{k \in \beta } \int _0^v (G_{\alpha \gamma }(s))_{ki }ds } dv \\&= - \ln \left( 1- \int _0^t \sum _{\beta \in X \setminus \{\alpha \} } \sum _{k \in \beta } (G _{\alpha \beta }(s))_{ki} ds \right) . \end{aligned}$$

Hence, $e^{- \int _0^t M_{\alpha i } (v) dv } = 1- \int _0^t \sum _{\beta \in X \setminus \{\alpha \} } \sum _{ k \in \beta } (G_{\alpha \beta }(s))_{ki } ds$. We deduce that ${\bar{G}}_{\alpha \beta } = G_{\alpha \beta }$. By uniqueness of the solution, we obtain $N_\alpha =\bar{N}_\alpha$. This concludes the proof. $\square$

### Initial conditions of ODEs compatible with a SPEs reformulation

We now characterize the initial conditions of the ODE (15) that guarantee that the corresponding forcing function $S^0_\alpha$ is of the form (79) for some $m_\alpha$. This allows to associate with these ODEs a SPE of the form (71)–(73). Indeed, starting from the ODEs (15) we can write a system of RFEs for $N_\alpha$, see Theorem 2.4. Then, if $S^0_\alpha$ is of the form (79) we can associate to this RFEs a SPEs. With this procedure we can therefore, implicitly, associate to these ODEs an age structure.

#### Proposition 6.7

(ODEs and SPEs) Let $N_\alpha$ be given by (12) where $\{n_\alpha \}$ is the family of solutions to (15). If for every $\beta \in X$ there exists a $m_\beta \in \left( {\mathcal {M}}_{+, b} ({\mathbb {R}}_+) \right) ^{|\beta |}$ such that

81 $$\begin{aligned} n^0_\beta = \int _{{\mathbb {R}}_- } e^{- \xi A_{\beta \beta }} m_\beta ( d\xi ) \end{aligned}$$

then $N_\alpha$ is given by (76), where $\{ f_{\alpha j } \}$ is the solution to a SPE of the form (71)–(73).

#### Proof

Theorem 2.4 implies that $N_\alpha$ satisfies Eq. (16). The flux $S_\alpha$ solves (17) with response functions $G_{\alpha \beta }(t)=A_{\beta \alpha } e^{t A_{\alpha \alpha }}$ and forcing functions

82 $$\begin{aligned} S^0_\alpha (t)= \sum _{\beta \in X \setminus \{ \alpha \}} G_{\beta \alpha } (t) n_\beta ^0 =\sum _{\beta \in X \setminus \{ \alpha \}} A_{\alpha \beta } e^{t A_{\beta \beta } } n_\beta ^0= \sum _{\beta \in X \setminus \{ \alpha \}} \int _{{\mathbb {R}}_-} G_{\beta \alpha } (t-\xi ) m_\beta (d\xi ), \end{aligned}$$

where in the last equality we used (81). As a consequence of (82) and Theorem 6.5 we can associate to the RFEs (16)–(18) a system of SPEs of the form (71)–(73). $\square$

As explained above, Proposition 6.7 guarantees that if (81) holds, then we can introduce an age structure in the ODEs system.

## Specific examples and applications: linear case

We now consider some examples of applications of the response function formalism in biochemistry. We consider first systems leading to linear problems. The examples that we study include the classical kinetic proofreading model introduced by Hopfield and Ninio (Sect. 7.1), a model of non-Markovian linear polymerization (Sect. 7.2), and a simple linear network inspired by the model of robust adaptation in Barkai and Leibler (1997), (see Sect. 7.3).

### Kinetic proofreading, Hopfield model

Our first application concerns a kinetic proofreading mechanism due to Hopfield Hopfield (1974) and Ninio Ninio (1975). The Hopfield model, or other mechanisms of kinetic proofreading inspired by that, have been found in many biological processes, including pathogen recognition from the immune system (Goldstein et al. 2004), McKeithan (1995), DNA replication, m-RNA translation, DNA recognition and DNA transcription, see the review (Boeger 2022) and also (Murugan et al. 2012; Pigolotti and Sartori 2016).

The classical Hopfield model is a biochemical network of reactions of the form

83 $$\begin{aligned} C \leftrightarrows S, \quad C \leftrightarrows S^* \end{aligned}$$

84 $$\begin{aligned} S \ \overleftarrow{ \rightsquigarrow } \ S^* \end{aligned}$$

85 $$\begin{aligned} C \rightarrow \emptyset , \quad S^* \rightarrow P \end{aligned}$$

where we indicate with $\leftrightarrows$ the reactions having detailed balance and with $\overleftarrow{ \rightsquigarrow }$ the reaction that does not have detailed balance. For more complex kinetic proofreading networks we refer to Murugan et al. (2012), Murugan et al. (2014).

It is important to notice that the state denoted by *S* is a complex that consists in the combination of the molecule denoted by *C* with some receptor. In the formulation of (83) and (84) we assume that the number of receptors is very large and therefore the number of attachment points for *C* can be assumed to be constant. On the other hand, $S^*$ is the phosphorylated state of *S* and it synthesize the product *P*. For example, *C* could be a codon on the mRNA, *S* the complex made of *C* and a tRNA. The product *P* would then be an amino acid. We refer to Chapter 7 in Alon (2019) for more biological details.

**Fig. 4:**
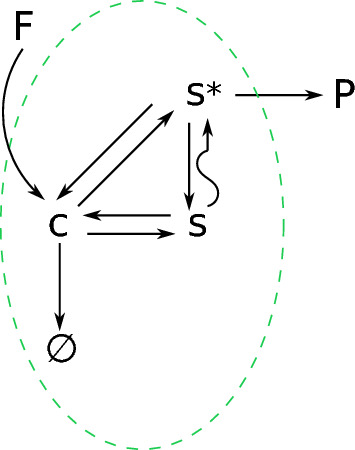
Reactions in the proofreading mechanism due to Hopfield. In the green dashed line, we have the compartment $\{C, S, S^*, \emptyset \}$

We assume that $$E(S)- E(C)=E_1 $$ while $$E(S^*) - E(C) = E_2 $$ where $$E(S), E(S^*), E(C) $$ are the free energies of *S*, $$S^*$$ and *C*. We stress here that we are choosing units of energy such that $$k_B T=1$$ where $$k_B$$ is the Boltzmann constant and *T* is the temperature of the system, so that the energies are dimensionless. The affinity $$E_1$$ to be detected by the receptor can be different for another molecule $${\overline{C}}$$, producing the complexes $${\overline{S}} $$ and $${\overline{S}}^*$$. In other words, the affinity $${\overline{E}}_1= E({\overline{S}}) - E({\overline{C}}) $$ can be slightly different from $$E_1 $$. Specifically, if the circuit has a preference for the molecule *C* over $${\overline{C}}$$ we have $${\overline{E}}_1 > E_1$$. However $$E_1-E_2$$ can be assumed to be constant, i.e. $$E_1-E_2= {\overline{E}}_1 - {\overline{E}}_2 $$ where $${\overline{E}}_2= E({\overline{S}}^*) - E( {\overline{S}}) $$. The reason why we can make this assumption is that the phosphorylation process takes place in a part of the molecule that is far from the part of the receptor where the molecule *C* or $${\overline{C}}$$ was attached. The phosphorylation reaction (84) does not have detailed balance due to the consumption of ATP (or energy) in an irreversible manner, see Fig. 4.

The rate of the reaction $$C \rightarrow S $$ is *k*. Due to the detailed balance condition the rate of the reaction $$S \rightarrow C$$ is $$k e^{E_1}$$. Indeed the detailed balance property guarantees that $$k N_C = k_- N_S $$ where $$k_-$$ is the rate of the reaction $$S \rightarrow C $$ and where $$N_C=e^{-E(C)}, N_S = e^{-E(S)}$$ are respectively the concentrations of *C* and *S* at the steady state. Therefore $$k_-= k \frac{N_C}{N_S} = k e^{E_1} $$.

Similarly the rate of the reaction $C \rightarrow S^*$ is $\beta$ and the one of the reaction $S^* \rightarrow C$ is $\beta e^{E_2}$. Finally the rate of the reaction $S \rightarrow S^*$ is $\alpha$. On the other hand, due to the lack of detailed balance, we will denote the rate of the reverse reaction $S^* \rightarrow S$ as $\alpha e^{E_2-E_1} /Q$, where *Q* is a coefficient which measures the lack of detailed balance. If $Q = 1$ the reaction (84) has detailed balance. On the contrary, if $Q>1$ energy is spent in an irrerversible manner to transform *S* into $S^*$.

More precisely, we consider the ODEs

86 $$\begin{aligned} \begin{aligned} \dfrac{dC}{dt}&= -(k+\beta +\mu )C+ke^{E_1} S +\beta e^{E_2} S^*+F(t) \\ \dfrac{dS}{dt}&= kC-(ke^{E_1}+\alpha ) S+\dfrac{\alpha }{Q}e^{E_2-E_1}S^* \\ \dfrac{dS^*}{dt}&= \beta C+ \alpha S-\left( \dfrac{\alpha }{Q}e^{E_2-E_1}+\beta e^{E_2} + \lambda \right) S^* \\ \dfrac{dP}{dt}&= \lambda S^*. \end{aligned} \end{aligned}$$

The equations for ${\overline{C}}, {\overline{S}}, {\overline{S}}^*$ are identical, except for the energies $E_1$ and $E_2$ which are ${\overline{E}}_1$ and ${\overline{E}}_2$. The term *F* in (86) is an external source of substance *C*.

Changing the time unit, we can assume without loss of generality that $k=1$ in (86). The effective behaviour of the reactions $C\rightarrow P$, $\bar{C}\rightarrow P$ can be described via the corresponding response functions $\Phi ,\, \bar{\Phi }$. The response functions $\Phi$ and ${\overline{\Phi }}$ can be obtained taking $F(t)=\delta _0(t)$.

Using the linearity and invariance under time translations of (86) we can rewrite the total production until the time *t* in the form

$$\begin{aligned} \frac{d}{dt}P(t)= \int _{-\infty }^t \Phi (t-s) F(s) ds \end{aligned}$$

where $\Phi (t) = \lambda S^*(t)$, where $S^*$ is computed solving (86) with initial value $C(-\infty ) = S(-\infty ) = S^*(-\infty )=0$ and $F(t)=\delta _0(t)$. This is equivalent to solving (86) for $t>0$ with initial conditions $C(0)=1$, $S(0)=S^*(0)=0$, $P(0)=0$, $F(t)=0$ for every $t >0$ respectively.

We will denote with $P_\infty$ the total quantity of product generated upon excitation by a signal $F(t)=\delta _0(t)$ of the molecule *C* and with ${\overline{P}}_\infty$ the same quantity produced by a signal of the molecule ${\overline{C}}$. Then, the total production ratio is given by

$$\begin{aligned} \frac{{\overline{P}}_\infty }{P_\infty }= \dfrac{\int _0^\infty \bar{\Phi }(t)\, dt}{\int _0^\infty \Phi (t)\, dt} = \dfrac{\hat{\bar{S}}^*(0)}{\hat{S}^*(0)}, \end{aligned}$$

where $\hat{\bar{S}}^*, \, \hat{S}^*$ are the Laplace transforms of $\bar{S}^*, \, S^*$. Taking the Laplace transform of (86), evaluating $z=0$ and solving the linear system leads to

$$\begin{aligned} \frac{{\overline{P}}_\infty }{P_\infty }=&\theta ^2 \, \dfrac{1+\beta +\beta \xi /\alpha \theta }{1+\beta +\beta \xi /\alpha } \, \dfrac{\xi ^2+\xi ((\beta +\mu )\lambda /\mu \beta \eta +\alpha /\beta Q+\alpha )+(1+\beta +\mu )\alpha \lambda /\mu \beta \eta }{\xi ^2+\theta \xi ((\beta +\mu )\lambda /\mu \beta \eta +\alpha /\beta Q+\alpha )+\theta ^2(1+\beta +\mu )\alpha \lambda /\mu \beta \eta } \\ \theta :=&e^{-(\bar{E}_1-E_1)}, \quad \xi := e^{E_1}, \quad \eta := e^{E_2-E_1}= e^{\bar{E}_2-\bar{E}_1}. \end{aligned}$$

Note that $\theta \in (0,1)$. Consequently, the above expression is greater than $\theta ^2=e^{-2(\bar{E}_1-E_1)}$. Let us mention that the optimal discrimination $\theta ^2$ agrees with the one originally obtained in Hopfield (1974) for constant flux solutions (i.e. assuming that *C* or ${\overline{C}}$ are constant in time, hence ignoring the first equation in (86)).

The value $e^{-2(\bar{E}_1-E_1)}$ is achieved if

87 $$\begin{aligned} \alpha \rightarrow 0, \quad \dfrac{\beta }{\alpha } \rightarrow 0, \quad \dfrac{\alpha }{\beta Q}\rightarrow 0, \quad \dfrac{\lambda }{\mu \eta }\rightarrow 0, \quad \dfrac{\lambda }{\beta \eta }\rightarrow 0, \quad \zeta :=\dfrac{\alpha \lambda }{\mu \beta \eta }\rightarrow 0 \end{aligned}$$

assuming that $\xi$ is of order 1. Notice that the first three limit expressions in (87) imply that $\alpha , \, \beta \rightarrow 0$ as well as $Q\rightarrow \infty$. The first two limits expression above imply that the reaction transforming *S* in $S^*$ is slow, but faster than the reaction transforming *C* in $S^*$. Furthermore, $Q\rightarrow \infty$ yields that detail balance is strongly violated in the reaction (84).

**Fig. 5:**
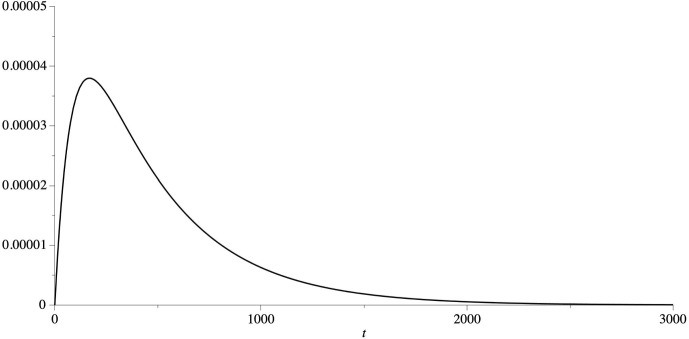
Plot of response function $\Phi (t)$ for $\alpha =\mu =\varepsilon$, $\beta =\varepsilon ^2$, $Q=1/\varepsilon ^2$, $\lambda =2\varepsilon ^2$, $e^{E_1}=2$, $e^{E_2}=4/\varepsilon ^s$, $e^{\bar{E}_1}=8$, $e^{\bar{E}_2}=16/\varepsilon ^s$ where $\varepsilon =0.01$, $s=1/2$

In order to ensure that the last three limits expressions in (87) hold we can either assume $\frac{\lambda }{\mu } \ll \frac{\beta }{\alpha }$ and $\lambda \ll \beta$ and $\eta$ of order 1. Alternatively, it is possible to obtain all the formulas in (87) also with $\eta \rightarrow \infty$, we refer to the examples later for this case. Note that $\mu \rightarrow 0$ is not required. However, if $\mu$ is of order one, most of the signal *C* will be lost in times of order 1 (or shorter) due to the term $-\mu C$ in (86).

In addition, we can also compute the total production $P_\infty$ under the assumptions (87). We then obtain

$$\begin{aligned} P_\infty&= \lambda \hat{S}^*(0) = \dfrac{1+\beta +\beta \xi /\alpha }{1+\beta +\mu +\xi (\mu \beta \eta /\lambda +\beta /\alpha +\mu /\alpha +\xi \mu \beta \eta /\lambda \alpha +\eta \mu /Q\lambda )}\\&= \dfrac{\zeta }{\xi ^2} \left( 1+o(1) \right) \end{aligned}$$

where $\zeta$ is defined as in (87) and goes to zero and $\xi$ is of order 1. We thus observe that the quadratic discrimination is obtained in the proofreading mechanism at the cost of having a very small fraction of molecules of *C* generating the product *P*.

We now consider specific forms of the response functions for some particular scaling limits of the chemical coefficients. More precisely, we set

88 $$\begin{aligned} \alpha =\varepsilon , \quad \beta =\varepsilon ^2, \quad Q=\varepsilon ^{-2},\quad \mu =\varepsilon , \quad \dfrac{\lambda }{\eta }= \varepsilon ^{2+s}, \end{aligned}$$

for some $s\in (0,1]$ and for $\varepsilon \rightarrow 0$. In this case, we have as $\varepsilon \rightarrow 0$

$$\begin{aligned} \frac{{\overline{P}}_\infty }{P_\infty } =\theta ^2 \left( 1+\mathcal {O}(\varepsilon ^s) \right) , \quad \frac{{\overline{P}}_\infty }{P_\infty } = \dfrac{\varepsilon ^s}{\xi ^2}\left( 1+\mathcal {O}(\varepsilon ^s) \right) . \end{aligned}$$

In Fig. 5 we plot the response function $\Phi$ in the case $s=1/2$, $\varepsilon =0.01$ and $\lambda =2\varepsilon ^{2}$, $\eta =2\varepsilon ^{-1/2}$ together with (88). Notice that the response function $\Phi$ that we obtain from this model is not an exponential function. This means that the dynamics is non-Markovian in general. However, as time tends to infinity $\Phi$ approaches an exponential function. This suggests that the system could be considered to be Markovian for times large enough.

**Fig. 6:**
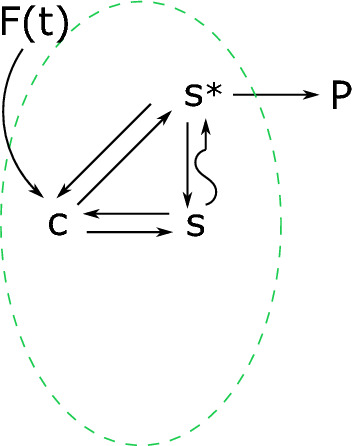
Reactions in the proofreading mechanism due to Hopfield, without the degradation of *C*

The degradation term $-\mu C$ in the equation for *dC*/*dt* is usually not included in the standard (time-independent) Hopfield problem as in Hopfield (1974), see Fig. 6. Here, since we consider a time-dependent model, the degradation term is needed to be able to discriminate, in terms of production of *P*, the molecule *C* and the molecule ${\overline{C}}$. Indeed, in the absence of degradation, every molecule *C* or ${\overline{C}}$ would synthesize the product *P*. Although, the molecule with more affinity will be much faster than the other one in the production of *P*, as we explain now. We compute the average time needed for the molecule *C* to produce *P*. This is given by

$$\begin{aligned} T= \frac{\int _0^\infty t \Phi (t) dt }{\int _0^\infty \Phi (t) dt } \end{aligned}$$

where $\Phi (t)= \lambda S^*(t)$ and where $S^*$ is computed from Eq. (86) with $\mu =0$ and with $C(0)=1$, $S(0 )=S^*(0 )=0$. Notice that, since $\frac{d}{dt} (C+S+S^*)= - \lambda S^*$, then

$$\begin{aligned} \int _0^\infty \Phi (t) dt= \lambda \int _0^\infty S^*(t) dt = C(0)=1, \end{aligned}$$

hence $$T=\int _0^\infty t \Phi (t) dt$$. Moreover,

$$\begin{aligned} T=\int _0^\infty t \Phi (t) dt= \lambda \int _0^\infty t S^*(t) dt = - \int _0^\infty t \frac{d}{dt} (C(t)+S(t)+S^*(t) )dt. \end{aligned}$$

Integrating by parts and using the fact that $C, S, S^*$ decay exponentially as time tends to infinity, we obtain that

$$\begin{aligned} T= \int _0^\infty (C(t)+S(t)+S^*(t) )dt = \hat{C}(0)+\hat{S}(0) + \hat{S^*}(0). \end{aligned}$$

Performing the Laplace transform in (86) we deduce that

$$\begin{aligned} T= \frac{( \lambda + \beta \eta e^{E_1} ) (k e^{E_1} +\alpha ) + \frac{\alpha }{Q} \eta k e^{E_1} + (k+\beta ) \frac{\alpha \eta }{Q}+k(\lambda +\beta \eta e^{E_1}) }{\lambda (\beta k e^{E_1} +\alpha k +\alpha \beta ) } \end{aligned}$$

We compare *T* with ${\overline{T}}$ under the following assumptions

$$\begin{aligned} \lambda \ll \beta \eta e^{E_1}, \quad \alpha \ll k e^{E_1},\quad \beta e^{E_1} \ll k,\quad \frac{\alpha }{Q} \ll \beta e^{E_1}, \quad e^{E_1} \gg 1 \end{aligned}$$

and deduce that

$$\begin{aligned} \frac{T}{{\overline{T}} } \approx e^{-2 (\bar{E}_1-E_1)}. \end{aligned}$$

This is in agreement with the results in Hopfield (1974) for solutions with constant fluxes.

### A linear polymerization model

In this section we describe a simple (linear) polymerization model which includes a kinetic proofreading mechanism. Polymerization processes are ubiquitous in biology. Some of the most important examples of polymerization in biochemistry are the transcription of DNA in mRNA and the translation of mRNA into polypeptides. Some of the most widely studied mathematical models of this last process can be found in MacDonald and Gibbs (1969), MacDonald et al. (1968), Pipkin and Gibbs (1966).

Most of the models of polymerization used in biology that we are aware of are Markovian. On the other hand, in all the polymerization models mentioned above kinetic proofreading mechanism and error correcting reactions take place whenever a monomer is incorporated to the polymer (see Pigolotti and Sartori (2016)). Hence, as explained in Sect. 7.1, it is natural to formulate a polymerization model for these processes in which the addition of monomers takes place in a non-Markovian manner. We will describe in this section a very simple polymerization model using the formalism of response functions to describe each polymerization step. Notice that we do not try to include in this model the simultaneous reading of a single mRNA strain by several ribosomes, as it has been done in MacDonald and Gibbs (1969), MacDonald et al. (1968), Pipkin and Gibbs (1966). Nevertheless we remark that the model considered here is the non-Markovian version of the model in Pipkin and Gibbs (1966).

We assume that polymers are characterized by their length $\ell$ and that polymers interact only with monomers. When a monomer binds to a polymer a sequence of kinetic proofreading reactions starts. After these reactions take place a monomer of size $\ell +1$ is formed. Due to the proofreading mechanisms the reaction $(\ell ) \rightarrow (\ell +1)$ is non-Markovian and, hence, we will model it using the formalism of response functions.

Let $n_\ell (t)$ be the number of polymers of length $\ell$ at time *t*. Then $n_\ell$ increases in time due to the flux $I_\ell$ of polymers from size $\ell -1$ to size $\ell$ and decreases due to the flux $I_{\ell +1}$ of polymers from size $\ell$ to size $\ell +1$. Namely, for $\ell \ge 1$, we have

89 $$\begin{aligned} \partial _t n_\ell (t) = I_\ell (t)- I_{\ell +1}(t), \quad \ell \ge 1 \end{aligned}$$

where

90 $$\begin{aligned} I_\ell (t)= \int _{-\infty }^t \Psi (t-s) I_{\ell -1 } (s) ds, \ \ell \ge 2. \end{aligned}$$

Here, to simplify the analysis, the response function $\Psi$ is assumed to be independent on $\ell$. Moreover, we assume that $\int _0^\infty \Psi (s) ds =1$.

We now study the long-time behaviour of $n_\ell$. To this end we assume that there is a constant flux of monomers entering the system, that is $I_1 (t) =1$ for all $t\ge 0$. Furthermore, we assume that $n_\ell (0)=0$ for every $\ell \ge 1$. Taking the Laplace transform to all the terms of Eq. (90) we deduce that

$$\begin{aligned} \hat{I}_\ell (z)= \hat{\Psi } (z) \hat{I}_{\ell -1} (z) \ \text { for } \ \ell \ge 2 \ \text { and } \ \hat{I}_{1} (z) = \frac{1}{z}. \end{aligned}$$

It follows that $\hat{I}_\ell (z)= \hat{\Psi }(z)^{\ell -1 }\hat{I}_1(z) = \frac{1}{z} \hat{\Psi }(z)^{\ell -1 }$. Applying the Laplace transform also to all the terms in Eq. (89), we deduce that $z \hat{n}_\ell (z)= \hat{I}_\ell (z) - \hat{I}_{\ell +1}.$ Hence

$$\begin{aligned} \hat{n}_\ell (z)= \frac{1}{z^2} \left( \hat{\Psi }(z)^{\ell -1} - \hat{\Psi }(z)^{\ell } \right) = \frac{1}{z^2} \hat{\Psi }(z)^{\ell -1} \left( 1 - \hat{\Psi }(z) \right) . \end{aligned}$$

In order to obtain the long-time asymptotics for $n_\ell$ we need to consider the asymptotics of $\hat{n}_\ell$ for *z* small. For *z* small the function $\hat{\Psi }(z)$ can be approximated by $1- \mu z$ where $\mu = \int _0^\infty s \Psi (s) ds$. Then

$$\begin{aligned} \hat{n}_\ell (z)= \frac{1}{z^2} \hat{\Psi }(z)^{\ell -1} \left( 1 - \hat{\Psi }(z)\right) \approx \frac{1}{z^2} (1-\mu z)^{\ell -1} \mu z = \frac{\mu }{z} (1-\mu z)^{\ell -1} \approx \frac{\mu }{z} e^{- \mu z \ell } \end{aligned}$$

as *z* goes to zero. Inverting the Laplace transform of $\frac{\mu }{z} e^{- \mu z \ell }$ we obtain that $n_\ell$ behave like a wave front of the form $n_\ell (t) \approx \mu \chi _{[\ell \mu , \infty )} (t)$ for large times. Notice that this solution describes a front of concentration in the space of polymer size propagating with speed $1/\mu$. A more detailed description of the solution near the edge of the front needs a more precise analysis that we will not pursue here.

### A linear chemical model of adaptation

An important concept in Systems Biology is the one of adaptation. A system shows adaptation if it reacts to gradients (in time or space) of a chemical, rather than to absolute values of each concentration. One of the earliest models of adaptation is the Barkai–Leibler model of bacterial chemotaxis, see (Alon 2019; Barkai and Leibler 1997). Other models of adaptation can be found in Ferrell (2016), Tang et al. (2010).

In this section we present a very simple model showing adaptation. This model can be thought as a linear version of the classical Barkai–Leibler model. This model is suited for a RFE reformulation as we have an input function, a compartment where reactions take place and an output. Although, in this case the definition of response function has to be slightly different from the one in the previous sections. Indeed, we will prove that the integral of the response function is equal to 0. This is necessary to induce adaptation as it forces the system to return to its initial state, if the signal remains constant for sufficiently long time.

The system of ODEs we consider is the following

$$\begin{aligned}&\frac{ d X}{dt} = a Y - b X + s(t) \\&\frac{ d Y}{dt} = 1- X \end{aligned}$$

with $a, b >0$. Here *X* measures the quantity of active receptors. It increases when the signal *s*(*t*) starts. Instead, *Y* is the response regulator protein. The output of this system will be the quantity of active receptors. We remark that the constant source term in the second equation could be the limit value of the Michaelis-Menten law in the saturation regime.

It is convenient to make the change of variables $\xi = X-1$ in the above equation, hence

91 $$\begin{aligned}&\frac{ d\xi }{dt} = a Y - b \xi - b + s(t) \nonumber \\&\frac{ d Y}{dt} = - \xi \end{aligned}$$

Notice that the steady state of *Y* is reached when $X={\overline{X}}=1$. We stress that ${\overline{X}}$ does not dependent on the signal *s*. This is a necessary property to have adaptation.

We now reformulate Eq. (91) using the formalism of response functions. We can think about $$\{ \xi , Y\}$$ as a compartment. The output of the chains of reactions taking place in the compartment is $$b\xi $$, while the input is $$s(t)-b $$. The dynamic inside the compartment $$\{ \xi , Y\}$$ is driven by the following subsystem of ODEs

$$\begin{aligned}&\frac{ d}{dt} \left[ \begin{matrix} \xi \\ Y \end{matrix} \right] = A \left[ \begin{matrix} \xi \\ Y \end{matrix} \right] \end{aligned}$$

where $$ A= \left[ \begin{matrix} - b & a \\ -1 & 0 \end{matrix}\right] . $$ We compute the response function $\Phi$. Applying Theorem 2.4 together with Lemma 2.6 we deduce that $\Phi (t)=( b, 0) e^{A t } e_1$.

Assuming that $b^2> 4 a$ we deduce that the matrix *A* has two real eigenvalues $\lambda _{\pm } = \frac{1}{2} \left( - b \pm \sqrt{b^2 - 4 a } \right)$, where $\lambda _-< \lambda _+ < 0$ corresponding to the eigenvectors

$$\begin{aligned} v_{\pm }= \left[ \begin{matrix} 1 \\ - \frac{\lambda _{\mp }}{a} \end{matrix}\right] . \end{aligned}$$

As a consequence $\Phi (t)= C_+e^{\lambda _+ t} + C_- e^{\lambda _- t}.$ Using the fact that $\Phi (0)=b$ we deduce that

$$\begin{aligned} C_+= \frac{ b \lambda _+}{\sqrt{b^2-4a}} \ \text { and } \ C_-= - \frac{ b \lambda _-}{\sqrt{b^2-4a}}. \end{aligned}$$

Hence, $\Phi (t)=\frac{b}{\sqrt{b^2-4a}} \left( \lambda _+ e^{\lambda _+ t} - \lambda _- e^{\lambda _- t} \right) .$ Then we have the following dependence of *X* on *s*(*t*)

$$\begin{aligned} X(t)= 1+ \dfrac{1}{b}\int _0^t \Phi (v) (s(t-v) - b ) dv. \end{aligned}$$

Notice that $$\int _0^\infty \Phi (v) dv =0$$. As a consequence, if $$s(t)\rightarrow k $$ as $$t\rightarrow \infty $$ with $$k >b $$, then $$X(t) \rightarrow 1$$ as $$t \rightarrow \infty $$. This form of the response function is the one that might be expected from a system exhibiting adaptation, i.e. the output converges to a constant value if the signal *s*(*t*) approaches a constant value.

## Specific examples and applications: non-linear case

In this section we present some examples of non-linear RFEs. We will not analyse the mathematics of the models presented here in full detail. The aim of this section is to explain how to apply the formalism of response functions in order to describe the interactions of different parts of a biochemical system, modelled by non-linear ODEs containing non-linearities of the form of mass action, or Michaelis-Menten. In particular, for these types of models it is possible to describe the response of a given compartment to a given set of inputs $I_i$ by means of functionals of the form

92 $$\begin{aligned} \begin{aligned} R(t)&= \sum _{i} \int _{-\infty }^t I_i(s)\psi _i(t-s) ds \\&\quad + \sum _{i} \sum _{j} \int _{-\infty }^t \int _{-\infty }^t I_i(s_1) I_j(s_2) \psi _{ij}(t- s_1, t- s_2 ) ds_1 ds_2 \\&\quad + \sum _{i} \sum _{j} \sum _k \int _{-\infty }^t \int _{-\infty }^t \int _{-\infty }^t I_i(s_1) I_j(s_2) I_k(s_3) \psi _{ijk}(t- s_1, t- s_2 , t-s_3) ds_1 ds_2 ds_3\\&\quad + \dots . \end{aligned} \end{aligned}$$

The main new feature of the non-linear models (92) compared to the linear ones of the form (1) is that the response can contain information about the correlations in time of the inputs $I_i$.

It is interesting to mention that RFEs of the form (92) containing only linear and quadratic terms of the inputs can yield a much richer set of responses than the linear RFEs. In Sect. 8.1 we show that one of the most common network motifs appearing in metabolic networks, Alon (2019), yields a response function of the form

$$\begin{aligned} R(t)&= \sum _{i} \int _{-\infty }^t I_i(s)\psi _i(t-s) ds \\&\quad + \sum _{i} \sum _{j} \int _{-\infty }^t \int _{-\infty }^t I_i(s_1) I_j(s_2) \psi _{ij}(t- s_1, t- s_2 ) ds_1 ds_2. \end{aligned}$$

Moreover, we will see that these type of response functions allow to describe the most distinguished features associated to this particular network motif.

Furthermore, in Sect. 8.2 we formulate equations that describe a non-Markovian polymerization model.

### Feed forward network

We can illustrate the usefulness of the theory of RFEs computing the RFEs for a specific biochemical network, namely the so-called Coherent Type 1 Feed Forward Loop (C1FFL). This network motif has been found often in many metabolic networks, we refer to Alon (2019) for an extensive description of this motif.

**Fig. 7:**
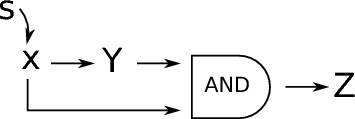
Type 1 coherent Feed Forward loop

We assume that a signal *S* activates a protein *X*, which promotes the production of a protein *Y*. Then the proteins *X* and *Y* jointly produce *Z*. This motif can be represented as in Fig. 7, using the logic formalism of the AND/OR-gates.

The main feature of the C1FFL is that it yields a delay in the production of *Z*, upon activation of the signal *S*. Instead, when the signals *S* stops, the production of *Z* stops immediately. Therefore, using the terminology of Alon (2019), this network is a sign-sensitive delay element. In this section, we will show a class of response functions exhibiting sign-sensitive delay. The biological advantage of this mechanism is that it avoids to produce an immediate response to a fast fluctuating input signal. We present now a system of ODEs representing the C1FFL and we examine the REFs type of equations that describe the relation between the input and the output of the system.

A possible way of modelling the C1FFL is with the following system of ODEs

93 $$\begin{aligned}&\frac{dX}{dt} = S- a X \nonumber \\&\frac{dY}{dt}= X-b Y \nonumber \\&\frac{d Z }{dt} = XY- c Z, \end{aligned}$$

where *a*, *b*, *c* are positive constants. From this system of equation we deduce that

94 $$\begin{aligned} X(t)= \int ^t_{- \infty } e^{-a (t-s) } S(s) ds, \quad Y(t)= \int _{-\infty }^t e^{- b (t-s)} X(s) ds. \end{aligned}$$

Using (94) we obtain

$$\begin{aligned} Y(t)&= \int _{-\infty }^t e^{- b (t-s)} X(s) = \int _{-\infty }^t e^{- b (t-s)} \int _{-\infty }^s S(v) e^{-a(s-v) } dv ds \\&= e^{-bt} \int _{-\infty }^t \int _v^t e^{s(b-a)} ds S(v) e^{ a v } dv = \frac{1}{b-a} e^{-at} \int _{-\infty }^t \left( 1 -e^{(v-t) (b-a)} \right) e^{ a v }S(v) dv. \end{aligned}$$

Finally, from the equation for *Z* we infer that

$$\begin{aligned} Z(t)&= \int _{-\infty }^t X(s) Y(s) e^{-c (t-s) } ds \\&= e^{-ct } \int _{-\infty }^t e^{ (c-2a)s } \left[ \int _{-\infty }^s e^{ a v } S(v) dv \right] \left[ \int _{-\infty }^s \frac{\left( 1- e^{(b-a)(w-s) }\right) }{b-a} e^{w a } S(w) dw \right] ds. \end{aligned}$$

Using Fubini, we deduce that

95 $$\begin{aligned} Z(t)= \int _{-\infty }^t \int _{-\infty }^t K(t- v, t- w) S(w) S(v) dw dv \end{aligned}$$

where

$$\begin{aligned} K(\eta , \xi ) = \int _{- \min \{\eta , \xi \}}^0 e^{(c-2a)s } e^{- a(\xi + \eta )} \frac{\left( 1- e^{ -(b-a)(\xi + s) } \right) }{b-a} ds, \quad \xi , \eta \ge 0. \end{aligned}$$

**Fig. 8:**
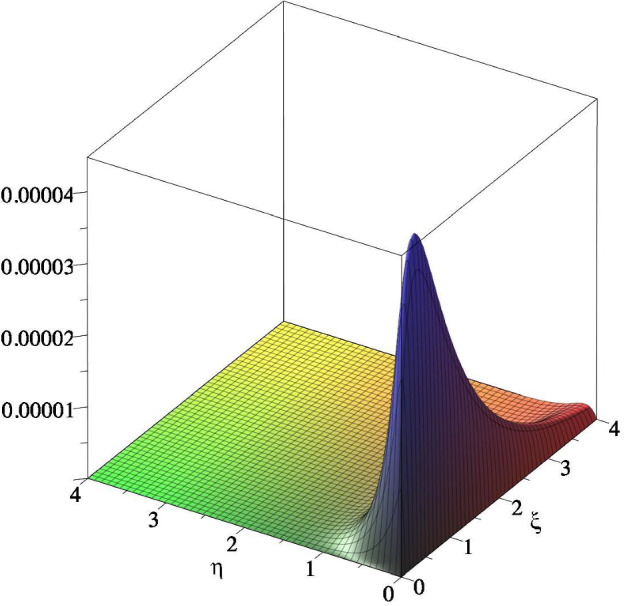
Plot of kernel $K(\eta ,\xi )$ in the case $a=c=5$, $b=1$

Note that

$$\begin{aligned} \frac{ 1- e^{ -(b-a)(\xi + s) }}{b-a} \ge 0 \end{aligned}$$

in the domain of integration. See Fig. 8 for a plot in the case $a=c=5$, $b=1$.

In order to gain insights on the RFE (95) we consider a limit situation. To this end, we assume that $a \gg 1$ and that $c \gg 1$. This means that we assume that the time scales 1/*a* and 1/*c* are much smaller than the characteristic time scale in which the signal *S* changes. Therefore, (93) implies that for $a \gg 1$ and $c \gg 1$

$$\begin{aligned} X(t) \approx \frac{1}{a} S(t), \ Y(t) \approx \frac{1}{a} \int _{-\infty }^t e^{-b(t-s) } S(s) ds \end{aligned}$$

and

$$\begin{aligned} Z(t)\approx \frac{1}{a^2c} S(t) \int _{-\infty }^t S(s) e^{-b(t-s)} ds. \end{aligned}$$

Assume that *S* has maximum value $S_m$. Then, the function *Z*(*t*) reaches values of the order of magnitude of its saturation value $S_m^2 /a^2 c b$ for times of order 1/*b*. This motivates to define the function ${\overline{S}}$ such that $S(t) = {\overline{S}} (tb)$ and to make the time rescaling $\tau = b t$. We also introduce the new variable $\xi (\tau )=a^2 bc Z(\tau ) / S_m^2$. Then for $\tau$ of order 1 we have

96 $$\begin{aligned} \xi (\tau )\approx {\overline{S}}(\tau )\int _{-\infty }^\tau {\overline{S}}\left( s\right) e^{-(\tau - s)} ds = \int _{-\infty }^\tau \int _{-\infty }^\tau K_0(\tau -s, \tau - v) {\overline{S}}\left( s\right) {\overline{S}}(v) ds dv \nonumber \\ \end{aligned}$$

where

$$\begin{aligned} K_0 (s_1, s_2)= e^{-s_1} \delta _0(s_2) \chi _{{\mathbb {R}}_*}(s_1). \end{aligned}$$

The kernel $$K_0 $$ is not symmetric, but we can rewrite (96) as

97 $$\begin{aligned} \xi (\tau )\approx \int _{-\infty }^\tau \int _{-\infty }^\tau \overline{K}_0(\tau -s, \tau - v) {\overline{S}}\left( s\right) {\overline{S}}(v) ds dv \end{aligned}$$

where

$$\begin{aligned} \overline{K}_0(s_1, s_2)= \frac{1}{2}\left( e^{-s_1} \delta _0(s_2) \chi _{{\mathbb {R}}_*}(s_1) + e^{-s_2} \delta _0(s_1) \chi _{{\mathbb {R}}_*}(s_2)\right) . \end{aligned}$$

We can examine now the response of the system to two signals, namely to ${\overline{S}}_1(\tau ) =\chi _{[0, \infty )} (\tau )$ and ${\overline{S}}_2 (\tau ) = \chi _{(-\infty , {\overline{t}}]} (\tau )$ with ${\overline{t}} >0$. Some care is needed to consider signals that are characteristic functions. In practise, the signals ${\overline{S}}_1$ and ${\overline{S}}_2$ must be understood as functions that change their values in time scales that are much larger than 1/*ba* and 1/*bc*. Notice that the response to the signal ${\overline{S}}_2$ yields $\xi (\tau )=0$ for every $\tau > {\overline{t}}$. So when the signal ${\overline{S}}_2$ suddenly stops, the level of $\xi$ decays instantaneously. On the other hand, in the case of the signal ${\overline{S}}_1$ $\xi$ reaches its saturation 1 after times $\tau$ of order 1. This is the expected behaviour for the C1FFL system.

We conclude this section by stressing that most of the network motifs in Alon (2019) can be formulated and analysed using non-linear RFEs including terms of the form (11), as we did in this section for the C1FFL.

### A nonlinear polymerization model

We formulate now a non-Markovian, non-linear polymerization model that has some analogies with the classical Becker-Döring equations. The Becker-Döring equations are a classical polymerization model that has been extensively studied as a model of phase transitions (see for instance (Hingant and Yvinec 2017)). The main difference between our model and the classical polymerization models is that in our case the addition of a monomer takes place in a non-Markovian way.

More precisely, we consider a population of polymers which can have different clusters sizes $\ell$. As in Sect. 7.2, we assume that polymers grow due to attachment of a monomer and we assume that the reaction $(\ell ) +(1) \rightarrow (\ell +1)$ is non-Markovian. More precisely, we assume that, when a monomer and a polymer of size $\ell$ bind, they do not form immediately a polymer of size $\ell +1$. Instead we assume that the addition of a monomer takes place by means of a chain of reactions that we do not try to model in detail, but that we represent with RFEs. Each of the reactions inside the chain can be assumed to be Markovian, but, as we have extensively seen in this paper, the relation between the inputs (in this case a monomer of size $\ell$ and one of size 1) and the output (which in this case is a monomer of size $\ell +1$) is typically non-Markovian and should be described using the formalism of response functions in this setting.

The main assumption of this model is that there exists an intermediate state after the attachment of a monomer to a polymer of size $\ell$ that describes the transient state until the polymer can be considered to have size $\ell +1$. We denote the density of these intermediate states with $w_\ell$. The equations describing the dynamics of the system of polymers is the following

$$\begin{aligned}&\frac{d n_\ell }{dt} = - n_1 n_\ell + n_{\ell +1 }-n_\ell + I_\ell \\&\frac{d w_\ell }{dt} = n_1 n_{\ell -1} - I_\ell \\&\frac{d n_1}{dt }= 2 n_2 - \sum _{\ell =2 }^\infty n_1 n_\ell - 2 n_1^2+ \sum _{\ell =2}^\infty n_{\ell +1} + S , \end{aligned}$$

where *S* is a source of monomers. The flux $I_\ell$ is given by

$$\begin{aligned} I_\ell (t)= \int _{-\infty }^t \Psi (t-s) n_1(s) n_{\ell -1}(s) ds \quad \ell \ge 2. \end{aligned}$$

Notice that $$S= \frac{d}{dt} \left( \sum _{\ell =1}^\infty \ell n_\ell + \sum _{\ell =2 } \ell w_\ell \right) $$.

In contrast with the model described in Sect. 7.2, here we have that the influx of polymer of size $$\ell $$ depends on the history of the number of monomers of size $$\ell $$ and of size 1, namely on all the values of $$n_\ell $$ and $$n_1 $$ in the time interval $$(- \infty , t]$$.

## Conclusions

In this paper we propose to use the formalism of RFEs to model complex biochemical systems. As explained in this paper, the interactions between different parts of biochemical systems can be non-Markovian. Since the RFEs can be thought as non-Markovian equations they are well suited to model these interactions. The formalism of RFEs has been already extensively used in biology, in the context of population dynamics and epidemiology (see for instance (Diekmann et al. 1998)).

In this paper we study under which conditions it is possible to reformulate a given model of ODEs as RFEs. We analyse mainly linear RFEs that are conservative, i.e. they conserve the total number of elements, although we consider also non-linear and non-conservative examples. Many applications in biology lead to non-linear models (see Sect. 8), or non-conservative models (see for instance (Zilman et al. 2010)). It would be interesting to extend the approach presented in this paper to these cases, as it has been done in Population Dynamics and Epidemiology.

Another possibility would be to consider space dependent response functions. This would allow to consider space dependent models. See for example (Galstyan et al. 2020) for an extension of the proofreading model that propose spatial gradients as a way to improve specificity.

As explained in Sect. 3, the structure of the biochemical reactions of the system impose constraints on the response functions. In particular we proved that systems satisfying the detailed balance condition are associated to completely monotone response functions. This opens the possibility of deriving the properties of biochemical systems from the (experimentally measurable) properties of the response functions.

Writing a model using RFEs allows to describe complex systems of reactions by means of some operators (linear or non-linear) that are characterised by a set of response functions. It is therefore relevant to determine if the behaviour of a biochemical system described by a specific system of ODEs can be captured by a REF with response functions having generic features (for instance that are completely monotone). This would allow to verify the robustness of the behaviour of the system as done in Population Dynamics and Epidemiology. An issue that is not considered in this paper is to describe the interactions between different biochemical circuits. This would require to consider a combination of many RFEs models.

We conclude by stressing that one of the questions addressed in this paper, on the relation between ODEs and RFEs, have some analogies with the one addressed in Diekmann et al. (2020) and in Diekmann and Inaba (2023) for models appearing in Population Dynamics and Epidemiology. The main difference is that in these papers are studied the conditions on the response functions (or kernels) of the renewal equation that allow a reformulation of the system as ODEs. Instead, in Sect. 2.3 we start from the ODEs models (well suited for systems of biochemical reactions) and we rewrite them using the RFEs formalism, that includes a renewal equation. As expected, the class of kernels we obtained with this procedure is the same one for which it is shown in Diekmann et al. (2020) and Diekmann and Inaba (2023) that a reformulation of the REs as ODEs is possible. In particular, it is the class of kernels that correspond to Markovian interactions between the compartments, see Sect. 4.
